# *Coxiella burnetii* inhibits host cell death by ubiquitination‐induced inactivation of an apoptosis nuclease

**DOI:** 10.1002/mlf2.70093

**Published:** 2026-08-27

**Authors:** Yong Zhang, Jun Jiao, Shuxin Liu, Jingya Yuan, Siyao Liu, Tao‐Tao Chen, Songying Ouyang, Jiaqi Fu, Zhao‐Qing Luo

**Affiliations:** ^1^ Department of Respiratory Medicine, Center of Infectious Diseases and Pathogen Biology, Key Laboratory of Organ Regeneration and Transplantation of The Ministry of Education, State Key Laboratory for Diagnosis and Treatment of Severe Zoonotic Infectious Diseases The First Hospital of Jilin University Changchun China; ^2^ State Key Laboratory of Pathogen and Biosecurity Beijing Institute of Microbiology and Epidemiology Beijing China; ^3^ Key Laboratory of Microbial Pathogenesis and Interventions of Fujian Province University, the Key Laboratory of Innate Immune Biology of Fujian Province, Biomedical Research Center of South China, Key Laboratory of OptoElectronic Science and Technology for Medicine of the Ministry of Education College of Life Sciences, Fujian Normal University Fuzhou China

**Keywords:** apoptosis, *Coxiella burnetii*, E3 ubiquitin ligase, effectors, type IV secretion system

## Abstract

*Coxiella burnetii* modulates a variety of host cell processes to create a niche permissive for its intracellular replication using effectors injected by its Dot/Icm type IV secretion system. More than 130 such effectors have been identified, but the function of most of them remains unknown. Using an activity‐based screening strategy, we identified the *C. burnetii* protein CubL1 (Cbu0295) as an E3 ubiquitin ligase. Further analysis reveals that CubL1 catalyzes ubiquitination of eIF3g, a dual‐function protein involved in apoptosis and protein translation initiation. CubL1‐induced monoubiquitination inhibits the nuclease activity of eIF3g, essential for its role in cell death execution. Host cells infected by a *C. burnetii* mutant lacking *cubL1* became more susceptible to cell death induction. Thus, *C. burnetii* employs multiple distinct mechanisms to maintain host cell viability to ensure successful completion of its intracellular life cycle.

## Impact statement

Maintaining host cell integrity is essential for the intracellular life cycle of bacterial pathogens, as it counteracts cell death signaling initiated by the host in response to infection. A number of effector proteins of the bacterial pathogen *Coxiella burnetii* inhibit host cell death, but the biochemical basis of such inhibition mostly remains elusive. Our discovery that CubL1 inhibits host cell death by catalyzing monoubiquitination of the terminal executioner nuclease eIF3g indicates that the bacterium targets multiple stages of cell death pathways. These results reveal important insights into mechanisms of both pathogenesis and signaling of host cells. The activity of CubL1 revealed by our study has laid the foundation for the development of novel anti‐virulence strategies to treat *C. burnetii* infections.

## INTRODUCTION

Detection of pathogen‐derived ligands or damage by specific receptors initiates various signaling pathways that ultimately leads to cellular events designed to prevent pathogen proliferation^1^. In scenarios in which such containment is not successful, infected cells often enact distinct cell death programs to terminate the infection by eliminating the site for pathogen replication^2^. Thus, inhibition of cell death is essential for successful infections, particularly those caused by obligate intracellular pathogens.

*Coxiella burnetii* is a highly infectious intracellular pathogen that causes Q fever, a flu‐like illness. Although the majority of cases of Q fever are asymptomatic or resolve spontaneously, acute infection carries the risk of progressing to serious chronic sequelae, most notably life‐threatening endocarditis, a condition that is notoriously difficult to treat^3^. *C. burnetii* poses a significant threat to public health due to its environmental stability, low infectious dose, and potential for large‐scale aerosol transmission. These factors have led to its classification as a category B select agent, highlighting the risk of its development as a bioweapon^4^, ^5^. Human infection with *C. burnetii* occurs primarily through the inhalation of contaminated aerosols originating from livestock^6^, ^7^. Bacteria phagocytosed by alveolar macrophages replicate in a membrane‐bound compartment termed *Coxiella*‐containing vacuole (CCV) that has features resembling those of lysosomes^8^, ^9^.

*C. burnetii* utilizes the Dot/Icm type IV secretion system to transfer effector proteins into host cells to modulate distinct cellular processes for the biogenesis of the CCV^8^, ^10^, ^11^. Among these, CvpB, CvpC, CvpD, and CvpE co‐opt distinct aspects of the vesicle trafficking pathway^12^. CvpF subverts RAB26‐dependent autophagy to promote vacuole biogenesis and virulence^13^. Two effectors have been found to inhibit NFκB signaling. NopA functions by targeting the Ran GTPase to block nucleus entry of NFκB^14^, whereas CinF employs a protein phosphatase activity to directly dephosphorylate IκB, thus allowing it to sequester NFκB in the cytoplasm to prevent effective immunity triggered by sensing the bacterium^15^. In addition, IcaA inhibits noncanonical activation of the NLRP3 inflammasome by an unknown mechanism^16^.

Multiple Dot/Icm effectors of *C. burnetii* are involved in suppression of cell death by diverse mechanisms^17^, ^18^. Among these, AnkG interferes with the host apoptosis pathway by interacting with the host protein gC1qR (p32)^17^; it also reprograms host cell transcription by modulating the 7SK small nuclear ribonucleoprotein complex^19^. CaeB inhibits endoplasmic reticulum (ER) stress‐induced cell death by promoting the nuclease activity of IRE1α, one key sensor of the unfolded protein response^20^. MceF localizes to mitochondria, where it recruits and hijacks the antioxidant glutathione peroxidase 4 to protect the host cell from oxidative stress‐induced cell death^21^.

Ubiquitin signaling regulates virtually every cellular process in eukaryotes, particularly immune responses, vesicle trafficking, and autophagy^22^. Many pathogens target the ubiquitin network for their benefit by virulence factors that function as E3 ligases with specific substrates^23^, and as enzymes that alter the activity of ubiquitin itself or components of the ubiquitination machinery by post‐translational modification^22^. A recent study found that EmcB of *C. burnetii* functions as a deubiquitinase to inhibit type I interferon production by removing ubiquitin chains from RIG‐I, the RNA sensor essential for interferon signaling^24^.

We herein explored *C. burnetii* Dot/Icm effectors potentially involved in ubiquitin signaling by an activity‐based screening, which identified CubL1 (Cbu0295) as an E3 ubiquitin ligase. Further study reveals that this bacterial E3 ubiquitin ligase induces ubiquitination of the eukaryotic translation initiation factor 3 subunit g (eIF3g), a dual‐function protein involved in both translation initiation and caspase‐mediated apoptosis as a DNase^25^. Ubiquitination of eIF3g prevents apoptosis of infected cells to allow productive bacterial replication.

## RESULTS

### Identification of Cbu0295 as an E3 ubiquitin ligase

**Figure 1:**
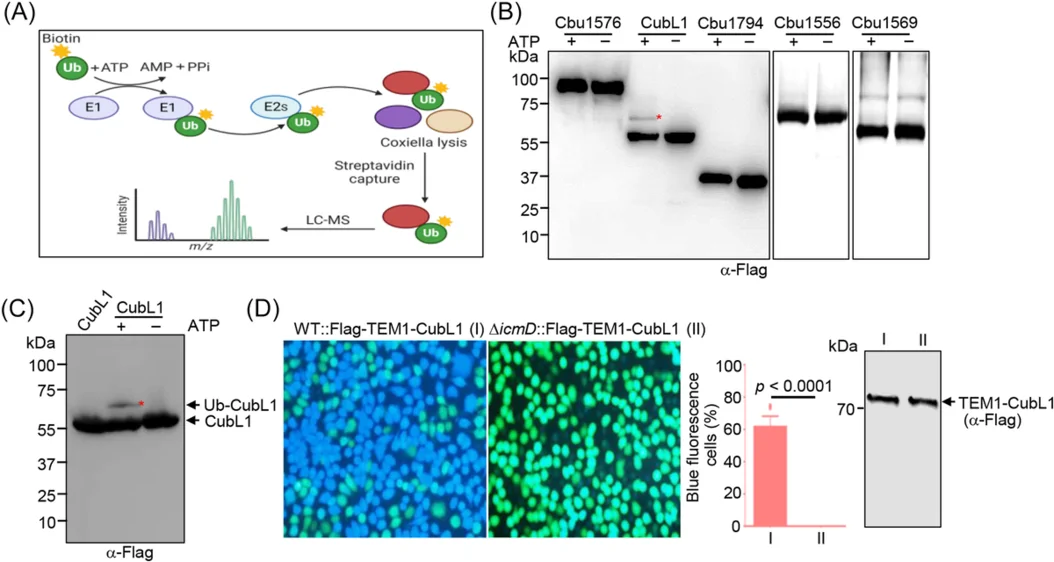
Identification of an E3 ubiquitin ligase from *Coxiella burnetii*. (A) A diagram of the procedure for the identification of E3 ubiquitin ligases from *C. burnetii*. Biochemical ubiquitination reactions were performed with E1, a combination of E2s, biotin‐labeled ubiquitin (Ub), and lysates of *C. burnetii*. Proteins modified by biotinylated‐Ub were enriched by streptavidin and identified by liquid chromatography‐mass spectrometry (LC‐MS) analysis. (B) Identification of E3 ubiquitin ligase from candidates obtained in (A) by biochemical ubiquitination assays. In each case, the Flag‐tagged candidate protein obtained from transfected mammalian cells was incubated with E1, a combination of E2s (Ube2R1, Ube2L3, Ube2D3, and Ube2G1), and Ub. Self‐ubiquitination of the candidate was determined by a shift in its molecular weight (MW) after probing by immunoblotting with the anti‐Flag antibody. (C) Further examination of the E3 ligase activity of CubL1. Reactions similar to those in (B) were established for CubL1 with an additional control in which only Flag‐CubL1 was present. Detection of ubiquitinated CubL1 was done as described in (B). (D) CubL1 is a substrate of the Dot/Icm system of *C. burnetii*. The indicated bacterial strains expressing Flag‐TEM1‐CubL1 were used to infect HeLa cells for 24 h, and samples were loaded with CCF4‐AM. Images were acquired using an IX‐83 fluorescence microscope 2 h after adding the substrate. Scale bar, 100 μm. The ratio of blue cells representing positive for protein translocation was determined by enumerating cells (*n* = 400) from three randomly taken images. Data (mean ± SEM) shown are from three independent experiments. The expression of the Flag‐TEM1‐CubL1 in *C. burnetii* was detected by immunoblotting with the Flag‐specific antibody. The results shown are representatives from two independent experiments with similar results.

To identify *C. burnetii* proteins with E3 ligase activity, we took advantage of the observation that many E3s are capable of self‐ubiquitination in biochemical reactions containing E1 and compatible E2 enzymes^26^. To this end, we established a series of reactions containing lysates of *C. burnetii*, recombinant E1, biotin‐labeled ubiquitin (Ub), ATP, and combinations of several E2 enzymes. Ubiquitinated proteins enriched with streptavidin‐coated beads were identified by liquid chromatography mass spectrometry (LC‐MS) analysis (Figures 1A and S1A). A total of 1136 proteins were obtained, among which 59 had been shown to be substrates of the Dot/Icm transporter by earlier studies (Table S1). To verify the E3 ligase activity of these candidates, we expressed Flag‐tagged proteins in HEK293T cells and isolated each protein by immunoprecipitation with beads coated with Flag‐specific antibody. Proteins eluted with Flag peptides were used to assay for E3 ligase activity, which revealed that Cbu0295 underwent self‐ubiquitination characterized by the formation of protein species of higher molecular weight (MW) (Figure 1B). The potential E3 ligase activity of Cbu0295 was further examined by experiments with samples containing only Flag‐Cbu0295 as controls (Figure 1C). Because of its potential E3 ligase activity, we referred to this effector as Coxiella ubiquitin ligase 1 (CubL1).

CubL1 has been shown to be a substrate of the Dot/Icm system, but the translocation efficiency appeared low (about 1%)^27^, we thus used the TEM‐1‐CCF4‐AM reporter system^28^ to revisit Dot/Icm‐dependent secretion by infecting HeLa cells with relevant *C. burnetii* strains that expressed the TEM1‐CubL1 fusion. Approximately 63% of cells infected with the wild‐type strain expressing TEM1‐CubL1 emitted blue fluorescence signals. In contrast, no cells in samples infected with the ∆*icmD* mutant expressing the same fusion produced blue fluorescence signals (Figure 1D). These results suggest that CubL1 is a substrate of the Dot/Icm system of *C. burnetii*.

### Biochemical properties of CubL1 and identification of its preferred E2

**Figure 2:**
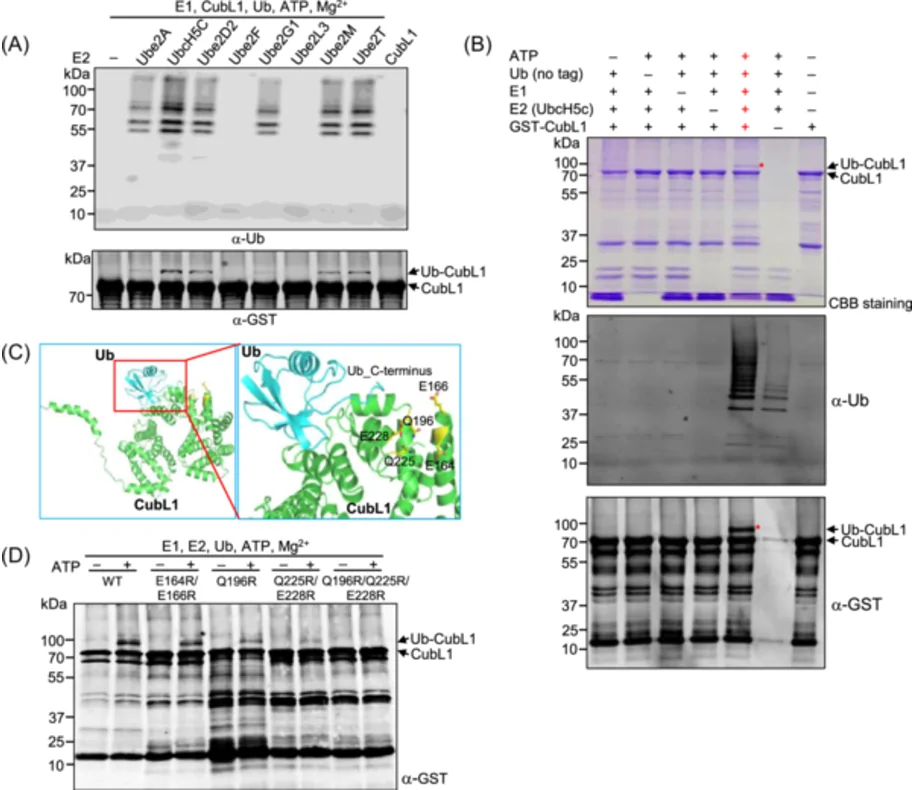
Identification of a preferred E2 for CubL1 and generation of catalysis‐defective mutants. (A) Self‐ubiquitination by CubL1 in reactions containing the indicated E2 enzymes. The reactions were allowed to proceed for 2 h, and modified proteins were detected using a Ub‐specific antibody. (B) Assessment of the E3 activity of CubL1 by dropout experiments. A series of reactions containing the indicated reactants was allowed to proceed for 2 h; proteins resolved by sodium dodecyl sulfate–polyacrylamide gel electrophoresis (SDS‐PAGE) were detected by Coomassie brilliant blue (CBB) staining (top) or immunoblotting with antibodies specific for Ub (middle) or GST (lower). (C, D) Identification of CubL1 residues essential for its E3 ligase activity. The interface between CubL1 and Ub predicted by molecular modeling (C) and the test of mutants for E3 ligase activity are shown. The indicated mutants were used in reactions containing E1, UbcH5C, and GST‐tagged CubL1 or its mutants. Activity was assessed by the production of CubL1 species with a high MW after detection with GST‐specific antibody. Note that the mutant CubL1_Q196R/Q225R/E228R_ (CubL1_3R_) was completely defective in self‐ubiquitination.

Bacterial E3 ligases exert their function by co‐opting the host ubiquitination machinery, particularly E2 conjugation enzymes, which play important roles in events such as catalysis and substrate selection^29^. We used a series of biochemical reactions, each containing an E2 of different classes, to identify the preferred E2 for CubL1. Self‐ubiquitination was detected in reactions receiving UbcH5C, Ube2D2, Ube2G1, Ube2M, or Ube2T, among which those containing UbcH5C displayed the most robust activity. No self‐modification was detected in reactions containing Ube2F or Ube2L3 (Figure 2A).

We next used UbcH5C to further establish the E3 ligase activity of CubL1 by a standard dropout experiment. Self‐ubiquitinated CubL1 was detected only in reactions containing all components required for canonical ubiquitination. When the products were detected by Coomassie brilliant blue (CBB) staining, a protein band potentially representing monoubiquitinated CubL1 was present in samples with complete reactants, which was in agreement with results from detection of ubiquitinated GST‐CubL1 by immunoblotting (Figure 2B). To further confirm that the signals of MW shift detected by antibodies specific for GST or Ub were due to ubiquitination, we added SdeA_1–200_, a potent deubiquitinase with relatively low chain type selectivity,^30^ to the reaction products catalyzed by CubL1. Such treatment eliminated the ubiquitination signals detected by antibodies specific for GST (by detecting MW shift) or Ub (Figure S1B). To determine whether host cells contain deubiquitinases capable of removing Ub from CubL1, we incubated Ub‐CubL1 with lysates of HEK293T cells. The results revealed that endogenous host DUBs can efficiently remove the ubiquitin moiety from self‐modified CubL1 (Figure S1C).

Next, we analyzed the E3 ligase activity of CubL1 by identifying residues critical for catalysis. Although their mechanism of action varies, many E3 ligases utilize an invariant cysteine (Cys) for catalysis, which is exemplified by members of the homologous to E6AP C‐terminus (HECT) family E3s^31^ and noncanonical E3s, such as SidC and SdcA, from *Legionella pneumophila* ^32^. We thus created a series of CubL1 mutants each with one of its seven Cys residues being replaced with alanine, purified recombinant proteins and assayed for E3 ligase activity. All of these mutants remained active at a level comparable to wild‐type protein (Figure S2), suggesting that CubL1 does not employ a Cys‐mediated catalysis.

To obtain catalytically inactive mutants, we sought to identify CubL1 residues potentially involved in Ub recognition by modeling its interactions with Ub using structures predicted by AlphaFold^33^. These efforts revealed that Glu164, Glu166, Gln196, Gln225, and Asp228 may make direct contact with Ub via hydrophobic interactions or formation of hydrogen bonds (Figure 2C). Based on the locations and proximity of the residues predicted to be important for Ub interactions, we constructed three mutants, CubL1_E164R/E166R_, CubL1_Q196R_, and CubL1_Q225R/E228R_. Among these, CubL1_Q196R_ and CubL1_Q225R/E228R_ displayed reduced E3 ligase activity (Figure 2D). We further examined the importance of these residues by creating a mutant in which Q196, Q225, and E228 were all substituted with an Arg residue. This mutant, termed CubL1_3R_, has completely lost the E3 activity in self‐modification assays (Figure 2D).

### The multifunctional protein eIF3g is a substrate of CubL1

**Figure 3:**
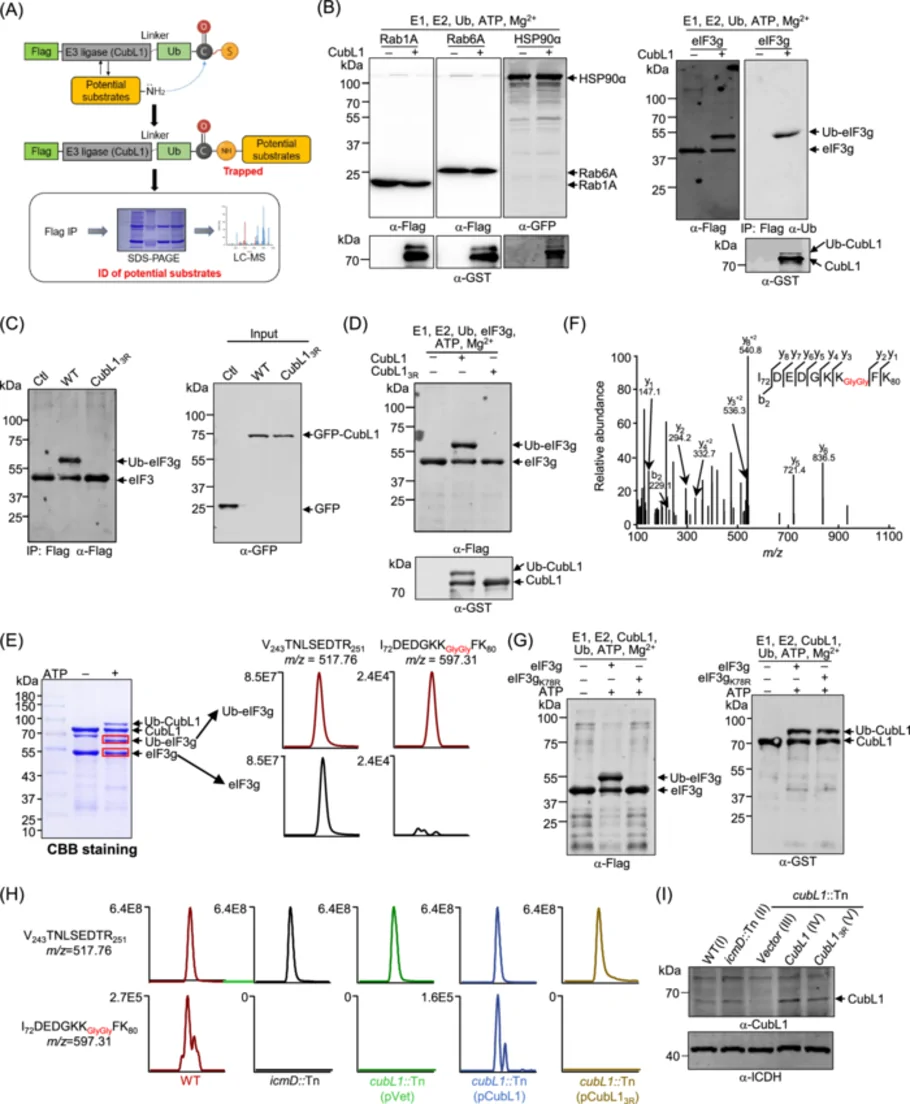
The multifunctional protein eIF3g is the cellular target of CubL1. (A) A diagram of the UBAIT‐based strategy to identify substrates of CubL1. After expression in HEK293T cells, the fusion proteins potentially linked to substrates were purified by immunoprecipitation with beads coated with the Flag antibody. Proteins were identified by LC‐MS analysis. (B) Screen of CubL1 substrates from candidates obtained in (A). Flag or GFP fusion of the indicated proteins isolated from transfected HEK293T cells was incubated with GST‐CubL1 and other components for ubiquitination for 2 h. Proteins were detected by immunoblotting using antibodies specific for GFP or GST. Ubiquitination was determined by the formation of protein species with an MW higher than their predicted sizes. (C) Ubiquitination of eIF3g by CubL1 *in cellulo*. HEK293T cells were transfected to express Flag‐eIF3g and GFP‐tagged CubL1 or the CubL1_3R_ mutant. Precipitates obtained by Flag antibody beads were probed with the Flag‐specific antibody (left panel). Expression of the GFP fusions was detected by immunoblotting with GFP antibodies. (D) Ubiquitination of eIF3g by CubL1 in biochemical reactions. Reactions containing Flag‐eIF3g and the indicated components were allowed to proceed for 2 h at 37°C. Modified Flag‐eIF3g was detected by immunoblotting using Flag‐specific antibody. (E–G) Identification of the ubiquitination site on eIF3g. Products of ubiquitination reactions containing GST‐CubL1 and GST‐eIF3g purified from *Escherichia coli* resolved by SDS‐PAGE were detected by CBB staining, and the protein bands representing modified and unmodified eIF3g were used to identify the ubiquitination site by LC‐MS analysis, respectively. A tryptic peptide harboring the Lys‐ϵ‐Gly‐Gly (diGly) remnant was detected in the upshifted band but not in the band migrated at the predicted MW (E). The spectrum for the ‐I_72_DEDGKK_GlyGly_FK_80_‐ fragment is shown in (F). Proteins of eIF3g and eIF3g_K78R_ were used for CubL1‐catalyzed ubiquitination reactions (G). Ubiquitinated eIF3g was detected by immunoblotting using Flag antibody (G, left panel), and GST‐CubL1 was detected using GST antibodies. Note that self‐ubiquitination of CubL1 occurred in reactions containing eIF3g_K78R_ even though the substrate was not modified (G, right panel). (H, I) Ubiquitination of eIF3g by CubL1 in cells infected by *C. burnetii*. The indicated *C. burnetii* strains were used to infect THP‐1 cells stably expressing Flag‐eIF3g for 3 days. Flag‐eIF3g purified by immunoprecipitation was analyzed by LC‐MS analysis to identify the ubiquitinated tryptic peptide‐I_72_DEDGKK_GlyGly_FK_80_‐ (H). The expression of CubL1 in these strains was probed by immunoblotting, and the metabolic enzyme isocitrate hydrogenase (ICDH) was probed as a loading control (I).

Owing to the low binding affinity between E3 ligases and their protein substrates, identification of proteins modified by a given E3 has always been a challenge^34^. We thus employed the ubiquitin‐activated interaction traps (UBAITs) method, which used an E3‐Ub fusion to trap the substrate^35^. To this end, we transfected HEK293T cells to express the Flag‐CubL1‐Ub chimera (Figure 3A). Total protein of 2 × 10^7^ transfected cells (from two petri dishes of confluent cells) was subjected to immunoprecipitation using beads coated with the Flag antibody, and bound proteins were resolved by sodium dodecyl sulfate–polyacrylamide gel electrophoresis (SDS‐PAGE). After CBB staining, proteins in bands that migrated at positions above the Flag‐CubL1‐Ub fusion (approximately 57 kDa) were identified by LC‐MS (Figure 3A). These efforts led to the identification of several candidate substrates, including Rab1A, Rab6A, HSP90α, and eIF3g. Each of these proteins was purified from mammalian cells in a Flag‐tagged form after transient transfection and assayed for ubiquitination by CubL1. Ubiquitination only occurred in reactions containing eIF3g, which is a multifunctional protein involved in eukaryotic translation initiation and apoptosis^36^, ^37^ (Figure 3B). Consistently, Flag‐eIF3g co‐expressed with GFP‐CubL1 in HEK293T became ubiquitinated (Figure 3C). In biochemical assays with recombinant proteins purified from *Escherichia coli*, ubiquitination of eIF3g occurred in reactions containing CubL1 but not the CubL_3R_ mutant (Figure 3D). In these reactions, only proteins corresponding to monoubiquitinated eIF3g were observed. These results indicate that eIF3g is a substrate for CubL1.

Many E3 ligases undergo self‐ubiquitination^26^, whether such self‐modification is important for the activity toward their substrates is less studied. We thus attempted to address this question by determining the sites self‐modified by CubL1 via LC‐MS analysis of Ub‐CubL1 isolated from biochemical reactions (Figure S3A). These efforts revealed that K446 and K467 were the sites receiving Ub in CubL1 (Figure S3B). Next, we constructed the CubL1_K446R/K467R_ mutant and tested its activity in reactions containing eIF3g. Our results showed that while self‐ubiquitination did not detectably occur, the mutant could still catalyze ubiquitination on the substrate eIF3g comparably to that of the wild‐type protein (Figure S3C). Thus, self‐ubiquitination is not important for the E3 ligase activity of CubL1.

To investigate the potential interaction between CubL1 and Ub or UbcH5C, we performed surface plasmon resonance (SPR) analysis, which showed that CubL1 has a binding affinity for Ub and UbcH5C of 1.693 × 10^−5^ M and 2.772 × 10^−6^ M, respectively (Figure S4A,C). Additionally, the 3 R mutant of CubL1 exhibited weakened interaction with Ub compared to its wild‐type counterpart (Figure S4B).

To further analyze CubL1‐catalyzed ubiquitination, we analyzed eIF3g modified by this bacterial E3 enzyme purified from *E. coli* by LC‐MS analysis, and found that Ub was linked to Lys78 of the protein (Figure 3E,F). In support of these results, the eIF3g_K78R_ mutant could no longer be ubiquitinated by CubL1 (Figure 3G). Importantly, self‐ubiquitination of CubL1 still occurred in these reactions, indicating that the inability to modify eIF3g in these reactions is due to the lack of a Ub acceptor site (Figure 3G, right panel).

Finally, we attempted to detect ubiquitinated eIF3g in cells infected with *C. burnetii*. To this end, we first performed transposon (Tn)‐induced mutagenesis^38^ of *C. burnetii* strain RSA493. A mutant in which the *cubL1* gene was inactivated by Tn at nucleotide site 107 was fortunately obtained from the first 24 candidates screened. Next, we established a stable cell line expressing Flag‐eIF3g from THP‐1 cells, which was infected with relevant *C. burnetii* strains for 3 days. Flag‐eIF3g was isolated from infected cells by immunoprecipitation, and ubiquitination was detected by LC‐MS analysis. The signal intensity of the reference peptide, V_243_TNLSEDTR_251_, was indistinguishable among the samples, suggesting that the amount of Flag‐eIF3g isolated was similar. Importantly, the tryptic peptide, I_72_DEDGKK_GlyGly_FK_80_, bearing the diGly remnant was detected in samples infected with the wild‐type *C. burnetii* strain but not with the *icmD*::Tn or the *cubL1*::Tn mutant (Figure 3H). Complementation of the *cubL1*::Tn mutant with CubL1 restored its ability to induce eIF3g ubiquitination during infection. In contrast, expression of the CubL1_3R_ mutant in this strain did not restore such modification (Figure 3H). We also examined the expression of CubL1 in these strains, which revealed that CubL1 was expressed in the wild‐type and the *icmD*::Tn strains but not in the *cubL1*::Tn mutant. Furthermore, CubL1 and CubL1_3R_ were similarly produced in the complementation strains (Figure 3I). These results further establish eIF3g as a target of CubL1.

Given the important role of eIF3g in translation, we examined whether CubL1 affects *de novo* protein synthesis. To this end, we transfected 5 × 10^4^ HeLa cells to express GFP, GFP‐CubL1, or GFP‐SidI. SidI is an effector from *L. pneumophila* known to potently inhibit host protein synthesis by targeting eEF1A^39^. Protein synthesis was probed using a click chemistry method involved in the incorporation of an amino acid analog into the growing polypeptide. As expected, GFP‐SidI completely inhibited the incorporation of l‐homopropargylglycine (HPG) into proteins, as no fluorescence signal was detected. In contrast, cells transfected to express GFP‐CubL1 displayed fluorescence signals indistinguishably to those that expressed GFP (Figure S5). Thus, the impact of CubL1 on host protein synthesis, if any, is negligible.

### CubL1‐induced ubiquitination abolishes the DNase activity of eIF3g

The protein eIF3g is a dual‐function protein involved in protein translation initiation and apoptosis^25^. Acting in cell death, eIF3g is cleaved by activated Caspase‐3 or Caspase‐7 between Asp220 and Gly221 in the SLRD_220_G region. The released amino terminal fragment promotes apoptosis by cleaving genomic DNA after being translocated to the nucleus^25^.

**Figure 4:**
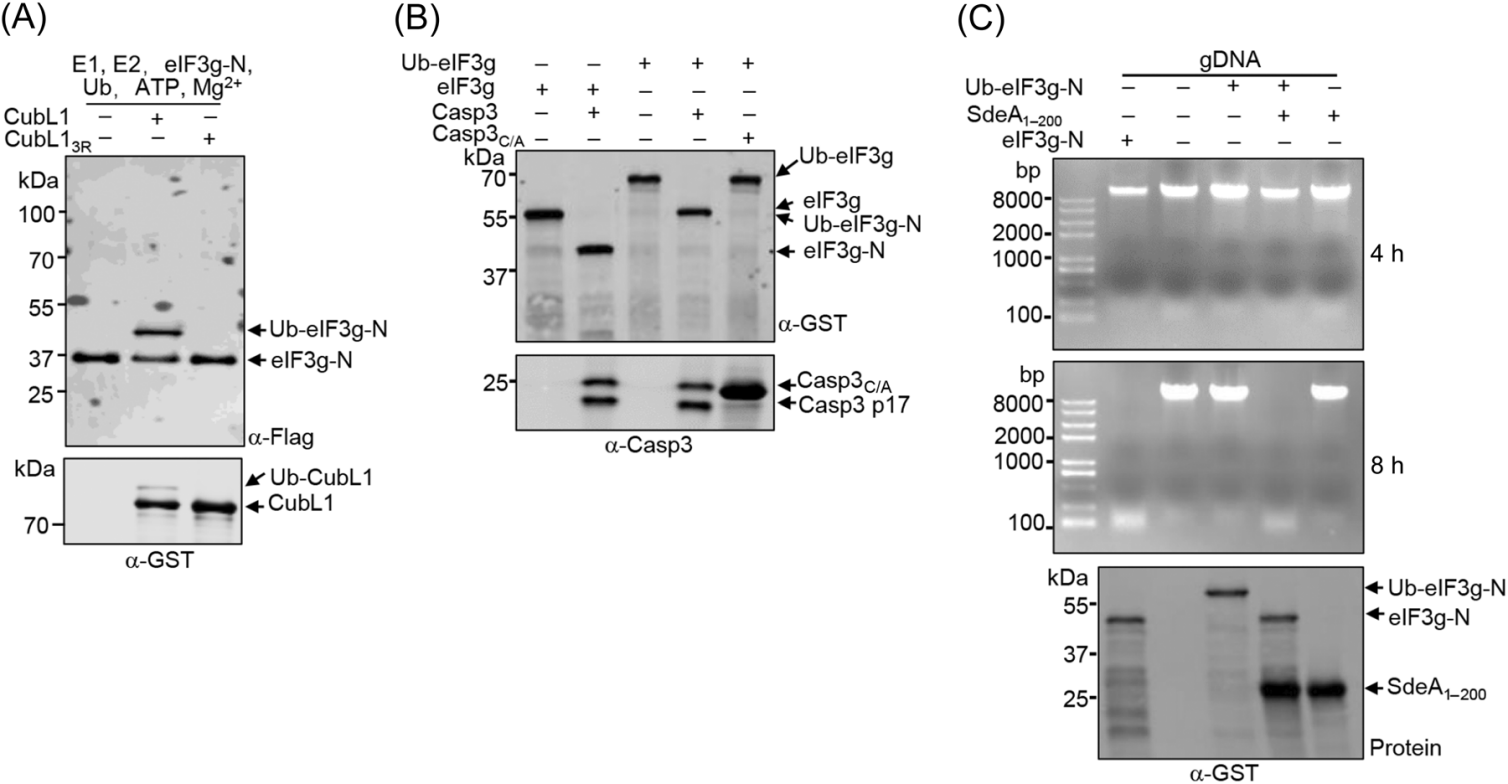
CubL1‐mediated ubiquitination abolishes the DNase activity of eIF3g. (A) The amino region of eIF3g‐N (1–230) can be ubiquitinated by CubL1. Reactions containing Flag‐eIF3g‐N and the indicated components were allowed to proceed for 2 h at 37°C. Samples resolved by SDS‐PAGE were probed by immunoblotting using antibodies specific for Flag (upper) or GST (for detection of GST‐CubL1). (B) Ubiquitinated eIF3g can still be cleaved by Caspase‐3 (Casp3). GST‐eIF3g or ‐GST‐Ub‐eIF3g was incubated with Casp3 for 2 h at 37°C. Reactions receiving a Casp3 mutant (Casp3_C/A_) defective in the protease activity were established as controls. Proteins separated by SDS‐PAGE were detected by immunoblotting using antibodies specific for GST or Casp3. Note that both native and ubiquitinated eIF3g were cleaved by Casp3. (C) Ub‐eIF3g‐N (1–230) is defective in digesting DNA. Genomic DNA of HEK293T cells was incubated in reactions containing eIF3g‐N or Ub‐eIF3g‐N (1–230) at 37°C for 4 and 8 h, respectively. The integrity of DNA was evaluated by agarose electrophoresis followed by ethidium bromide staining. To confirm that ubiquitination impacts the DNase activity, the deubiquitinase SdeA_1–200_ was added to a set of reactions containing Ub‐eIF3g‐N. Note that Ub‐eIF3g‐N could not degrade genomic DNA, but it regained the DNase activity after deubiquitination by SdeA_1–200_.

We first examined whether the cleavage affects the suitability of eIF3g as a substrate of CubL1. We produced a truncated version of eIF3g with DNase activity, which corresponds to the cleaved product (eIF3g‐N), and used it in ubiquitination reactions with CubL1. Robust modification of eIF3g‐N (1–230) occurred. Again, the CubL_3R_ mutant could not catalyze ubiquitination of this fragment of eIF3g (Figure 4A). Thus, the bacterial E3 recognizes both full‐length eIF3g and its cleavage product as substrate.

Next, we determined the effects of CubL1‐induced ubiquitination on its cleavage by Caspase‐3. Ub‐eIF3g purified from biochemical reactions (Figure S6) was incubated with recombinant Caspase‐3 or its enzymatically inactive mutant Caspase‐3_C/A_. Reactions containing native eIF3g were established as controls. Our results indicated that both native and ubiquitinated eIF3g could be cleaved by Caspase‐3. As expected, the Caspase‐3_C/A_ mutant could not cleave either form of eIF3g (Figure 4B).

To determine the impact of CubL1‐induced ubiquitination on the DNase activity of eIF3g, we incubated purified Ub‐eIF3g with human genomic DNA and examined its integrity by agarose gel electrophoresis and ethidium bromide staining. 2 μg of DNA was eliminated by 1 μg eIF3g‐N (1–230) after 8 h incubation. Such degradation did not occur in reactions receiving Ub‐eIF3g‐N (1–230) (Figure 4C). Importantly, removal of the ubiquitin moiety from Ub‐eIF3g using the deubiquitinase SdeA_1–200_ ^30^ restored its DNase activity. The integrity of DNA was not affected in reactions containing solely SdeA_1–200_ (Figure 4C). Moreover, structural modeling revealed that ubiquitinated lysine 78 (K78) of eIF3g localized within its nuclease active site region^25^ (Figure S7). This finding suggests that K78 ubiquitination may sterically inhibit the activity of eIF3g. To determine whether host cells contain deubiquitinases capable of removing ubiquitination from eIF3g mediated by CubL1, we incubated Ub‐eIF3g with cell lysates and deubiquitinases USP46 or USP5. None of these treatments led to the removal of Ub from modified eIF3g (Figure S8). Taken together, these results indicate that CubL1 targets both full‐length and the cleaved amino‐terminal portion of eIF3g by ubiquitination, and such modification inhibits its DNase activity.

### CubL1 functions to inhibit host cell death

**Figure 5:**
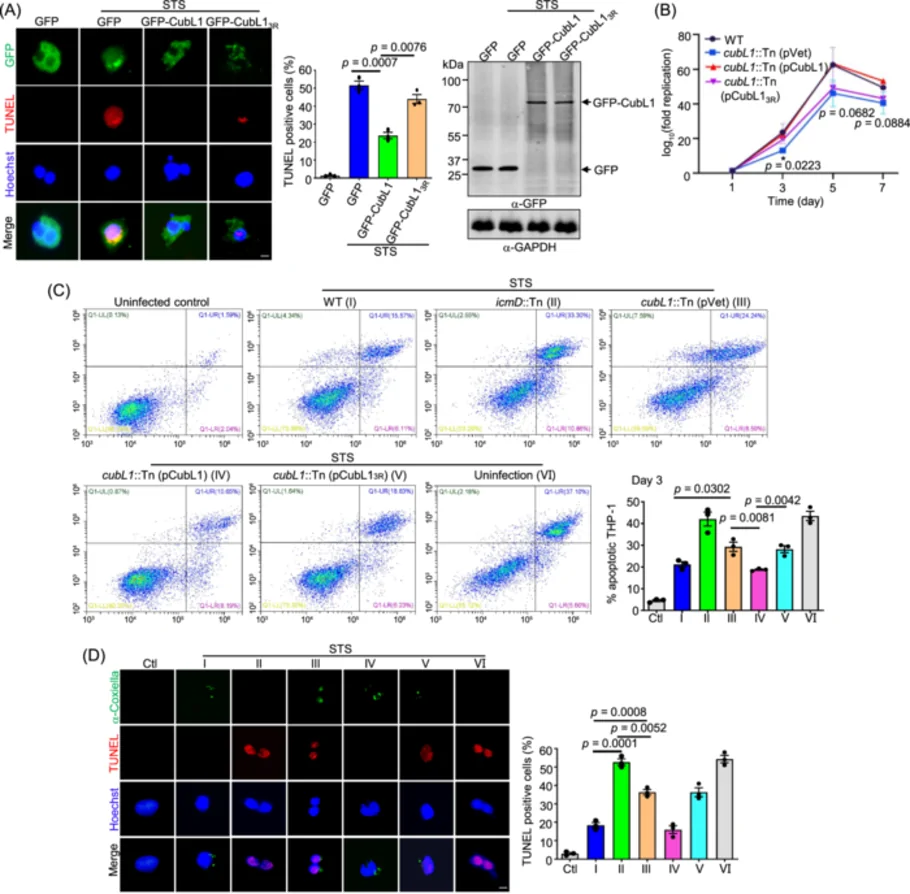
CubL1 inhibits host cell apoptosis in an E3 ligase activity‐dependent manner. (A) Cells expressing CubL1 are resistant to apoptosis induction. 5 × 10^6^ cells transfected to express GFP, GFP‐CubL1, or GFP‐CubL1_3R_ for 16 h were treated with 1 M staurosporine (STS, a protein kinase inhibitor) for 4 h. Fixed cells were stained with TUNEL reagents. Representative images (left) acquired using an IX‐83 Olympus fluorescence microscope and rates of apoptotic cells (mean ± SEM) (middle) are shown. Experiments were done in triplicate, and at least 300 cells from each sample were counted. Expression of the GFP fusions was probed by immunoblotting with GFP‐specific antibodies (right panel). Scale bar, 5 μm. (B) Bacterial replication of the indicated strains grown in ACCM‐D medium. The indicated strains were used to infect THP‐1 cells at a multiplicity of infection (MOI) of 200. Samples withdrawn at the indicated time points were determined for the genome equivalent to evaluate bacterial replication. Results (mean ± SEM) shown are from three independent experiments, each done in triplicate. (C, D) CubL1 contributes to resistance to STS‐induced cell death by *C. burnetii*. THP‐1 cells infected with the indicated bacterial strains for 3 days were treated with STS for 4 h, and samples were fixed and stained with the Annexin V‐FITC Apoptosis Detection Kit prior to determining the rate of Annexin V‐FITC‐positive cells by flow cytometry (C). Similarly, infected samples were stained with TUNEL reagent, and representative images of the samples and the rates of cell death (D) were determined as described in (A). Quantitative results (mean ± SEM) shown are from three independent experiments, each done in triplicate. Statistical analysis was performed by the *t*‐test. For TUNEL staining, at least 300 cells were scored in each sample. Scale bar, 5 μm.

The finding that CubL1‐induced ubiquitination abolished the DNase activity of eIF3g suggests that this E3 ligase inhibits cell death. We thus transiently expressed CubL1 or the CubL_3R_ mutant in HEK293 cells, treated the samples with staurosporine (STS, a protein kinase inhibitor), which induces apoptosis by inhibiting the activity of several kinases^40^. Evaluation of cell death by TUNEL staining revealed that in samples transfected to express GFP, with STS treatment for 4 h, caused approximately 50% cell death; the cell death rates dropped to about 20% in samples transfected to express GFP‐CubL1 (Figure 5A). Furthermore, expression of the CubL1_3R_ mutant defective in the E3 ligase activity failed to protect cells from apoptosis induced by STS (Figure 5A).

To probe the role of CubL1 in *C. burnetii* virulence, we first examined the impact of its inactivation on intracellular bacterial growth. We infected THP‐1 cells with the panel of *C. burnetii* strains (Figure 3I) and determined their replication. In THP‐1 cells, compared to the wild‐type strain, the *cubL1‐*Tn mutant displayed a significant defect in intracellular growth 3 days post infection (*p* = 0.0223), and this defect could be complemented by CubL1 but not the ligase‐defective CubL1_3R_ mutant (Figure 5B).

Given its ability to inhibit host cell death when expressed by transfection, we determined such a role under infection conditions using two sets of experiments. First, we infected 2 × 10^6^ cells with relevant *C. burnetii* strains for 3 days, and samples treated with 1 μM STS for 4 h were stained with Annexin V‐FITC prior to analysis by flow cytometry. Under our experimental conditions, samples left uninfected or infected with the *icmD* mutant contained about 45% apoptotic cells, and such rates were approximately 20% in samples infected with wild‐type *C. burnetii* (Figure 5C). Close to 32% of the cells infected with the *cubL1* mutant were positive for Annexin V‐FITC staining and expression of CbuL1, but not the CubL1_3R_ mutant in strain *cubL1*::Tn, reduced the cell death rates to about 20% (Figure 5C). Similar results were obtained when the apoptosis status of the infected cells was evaluated by TUNEL staining. More that 50% of cells infected with the *icmD*::Tn mutant were TUNEL positive. Such rates were about 20% in samples infected with the wild‐type strain (Figure 5D). In samples infected with the *cubL1*::Tn mutant, approximately 35% of the infected cells were apoptotic, which dropped to about 18% when the mutant was complemented by expressing CubL1. In contrast, expression of the ligase‐defective mutant CubL1_3R_ did not restore the ability of strain *cubL1*::Tn to confer resistance to STS‐induced cell death (Figure 5D). The role of CubL1 in protecting infected cells from apoptosis was extended to Day 4 after bacterial uptake, and the effects became less apparent on Day 5 (Figure S9).

## DISCUSSION

**Figure 6:**
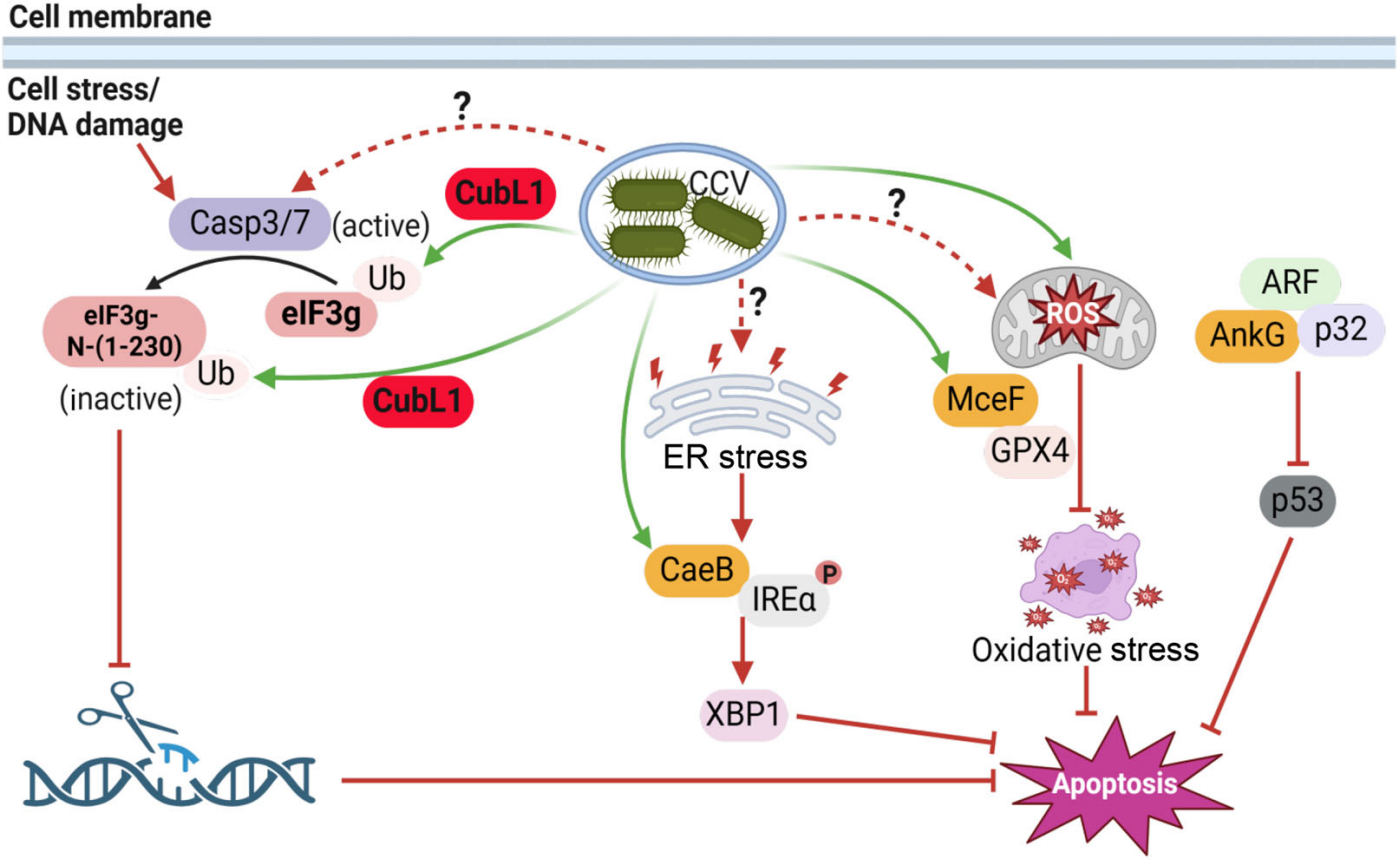
A model for the mechanisms of inhibiting host cell death by *C. burnetii*. By yet unknown mechanisms, infection by *C. burnetii* triggers host cell death by activating cascades of p53, ER stress, and ROS production, which are modulated by AnkG, CaeB, and MceF, respectively. CubL1 recognizes and ubiquitinates both the full‐length and Casp3‐cleaved eIF3g to inhibit cell death initiated by the intrinsic pathway involved in the activation of Casp3/7. CCV, *Coxiella*‐containing vacuole; ER, endoplasmic reticulum; ROS, reactive oxygen species.

Induction of cell death is an effective strategy for infected cells to eliminate or limit invading pathogens when other means to resolve the infection have failed. The death of host cells will be disastrous for obligate intracellular pathogens because their replication entirely depends upon viable cells. As a result, successful pathogens have acquired multiple often redundant mechanisms to block cell death triggered by the host in response to infection. The obligate bacterial pathogen *C. burnetii* represents one such example^41^. At least three Dot/Icm effectors have been shown to inhibit apoptosis triggered by intrinsic or extrinsic signals. Among these, AnkG appears to block apoptosis by multiple mechanisms. It interacts with and sequesters the mitochondrial pro‐apoptotic protein p32^42^, ^43^. In addition, nucleus‐localized AnkG induces transcription of cell survival genes by binding to both the DExD box RNA helicase 21 (DDX21) and the 7SK small nuclear ribonucleoprotein complex^19^. CaeA is also a nuclear protein that functions by preventing the cleavage of the executioner Caspase‐7^44^. CaeB blocks ER stress‐induced cell death by interacting with and upregulating the RNase activity of IRE1α^20^. In each case, the target protein of the effector is situated relatively upstream of the involved cell death pathway (Figure 6). Whether AnkG, CaeA, and CaeB exert their impact on host cell death by inducing post‐translational modification remains to be determined.

Acting in cell death, eIF3g causes direct damage by destroying genomic DNA after being activated by Caspase 3‐mediated cleavage^25^. The ability of CubL1 to ubiquitinate both full‐length and truncated forms of eIF3g (Figure 4) will provide better protection after cell death has been initiated in response to *C. burnetii* infection. Clearly, targeting a protein in the terminal position of the signaling cascade adds another layer of protection to ensure the viability of the infected cells (Figure 6). Akin to its inhibition of cell death, *C. burnetii* suppresses host immunity using redundant mechanisms by targeting different steps of an immune signaling cascade. For example, NopA and CinF inhibit the NF‐κB pathway by blocking nuclear translocation of RelA and IκBα dephosphorylation, respectively^14^, ^15^.

eIF3g is a dual‐function protein involved in protein translation and cell death^25^, ^45^. Expression of CubL1 by transfection did not detectably affect protein synthesis (Figure S4), suggesting that the monoubiquitination specifically inhibits the DNase activity without interfering with its role in protein translation. Alternatively, CubL1 may selectively target a pool of eIF3g that is specifically involved in cell death. Despite extensive efforts, we were unable to obtain recombinant CubL1 that exhibits more robust enzymatic activity. The protein may not be properly folded when expressed in *E. coli,* or the buffer system we used is not optimal for its catalysis. Under natural conditions, CubL1 injected into host cells by *C. burnetii* likely is more active, capable of modifying multiple target molecules prior to its decay, a phenomenon that has been documented for many bacterial effectors^46^.

Differing from the related bacterial pathogen *L. pneumophila*, which hijacks the host ubiquitin signaling by a large cohort of effectors with vastly diverse mechanisms^47^, EmcB is the only *C. burnetii* Dot/Icm effector currently known to be involved in ubiquitination; it interferes with anti‐bacterial interferon signaling by functioning as a deubiquitinase that removes Ub chains from the RNA sensor RIG‐I^24^. *C. burnetii* Dot/Icm effector genes often do not start to express after being engulfed by host cells^48^, ^49^, which may explain that CubL1 is the only E3 ubiquitin ligase identified in our activity‐based screens. Alternatively, some ligases may employ catalysis mechanisms that cannot be captured by our method. For example, an F‐box protein that requires SkpI and Cullin to form an active E3 ligase^50^ will likely escape our screen assays.

That ubiquitination by CubL1 occurs independent of a cysteine residue and suggests the use of a mechanism different from members of the HECT domain E3s (Figure S2)^51^ or noncanonical E3s of bacterial origins, such as SidC and SdcA, from *L. pneumophila* ^32^. No matter what the mechanism, the loss of catalysis by the CubL1_3R_ mutant suggests that direct contact with Ub is essential for its activity (Figure 2D–E). Further structural and biochemical studies are needed to elucidate its mechanism of catalysis. The potentially unique catalytic mechanism of CubL1 and its importance for optimal intracellular bacterial growth make it an attractive target for anti‐virulence‐based therapeutics. Such strategies are anticipated to exert minimal off‐target effects on host cellular processes.

A recent study shows that *C. burnetii* induces cell death at the late phase of infection to facilitate bacterial egress^52^, which suggests the need to lift the blockade in cell death as infection proceeds to the end. One salient feature of ubiquitination is its reversibility by specific enzymes. Metaeffectors that reversibly modulate the activity of host proteins have been well documented in *L. pneumophila* ^53^, ^54^, ^55^. It will be of great interest to determine whether *C. burnetii* uses metaeffectors to restore cell death execution for bacterial release from infected cells.

## MATERIALS AND METHODS

### Cell culture and transfection

HEK293T and HeLa cells, obtained from the ATCC, were cultured in Dulbecco's modified Eagle's medium (HyClone) supplemented with 10% fetal bovine serum (FBS) and 2 mM l‐glutamine at 37°C in a 5% CO_2_ incubator. THP‐1 monocytes were maintained in RPMI‐1640 medium containing 10% FBS under the same conditions. Plasmid transfections were performed using Lipofectamine 3000 (Thermo Fisher Scientific) according to the manufacturer's protocol. All cell lines were routinely tested for mycoplasma contamination using a PCR‐based kit (cat# MP0025; Sigma).

### Bacterial strains, infection, and protein translocation assay

All *C. burnetii* strains used in this study (Table S2) were derived from the Nine Mile phase II strain RSA493. The bacteria were cultivated, axenically in either liquid ACCM‐D medium or ACCM‐D agarose under microaerophilic conditions (37°C, 5% CO_2_, 2.5% O_2_)^56^, ^57^. When required, kanamycin and chloramphenicol were supplemented into the ACCM‐D medium at final concentrations of 300 and 3 μg/ml, respectively.

To examine protein translocation into host cells by *C. burnetii*, we constructed a plasmid that expresses the TEM1‐Flag‐CubL fusion by sequentially inserting the *TEM‐1* and *cubL1* genes into pJB‐Kan‐P1169‐3xFlag^58^. This plasmid, designated as pTEM1‐CubL1, was introduced into wild‐type and the *icmD*::Tn mutant of *C. burnetii* ^59^ by electroporation, respectively. The resulting strains were cultured in ACCM‐D medium for 5 days; 2 × 10^5^ HeLa cells were seeded in 24‐well plates without antibiotics for 12 h, and bacteria were added to cells at a multiplicity of infection (MOI) of 100. The plates were then centrifuged immediately at 250*g* for 10 min at 25°C to facilitate bacterial internalization. Infections were allowed to proceed for 24 h at 37°C in a CO_2_ incubator (5%). Cells were then washed three times with PBS, CCF4‐AM was added at a final concentration of 1 μM, and the samples were further incubated for 1 h. Protein translocation was examined for the emission of blue fluorescence signals by infected cells using an Olympus X‐83 fluorescence microscope. Images were acquired to determine the rates of Förster resonance energy transfer (FRET) in samples by counting the number of blue fluorescence cells.

### Genetic manipulation of *C. burnetii* and intracellular growth assays

Electrocompetent cells of *C. burnetii* were prepared using a previously described protocol^60^. Plasmids were introduced into the bacteria by electroporation using a Gene Pulse (Gene Pulser Xcell; Bio‐Rad) by the following parameters: 1.8 kV, 400 Ω, and 25 μF for 0.1 cm cuvettes.

To identify transposon‐induced mutants, strain RSA493 was mutagenized using the plasmid pKM225, which carries a Himar1 TnA7 transposase encoding chloramphenicol resistance^27^. Briefly, 5‐μl (10 μg) of pKM225 DNA was mixed with 50 μl of *C. burnetii* suspension. The cell suspension was placed in a 0.1‐cm gap electroporation cuvette on ice and electroporated as described earlier^61^. Following electroporation, bacteria were allowed to recover in 5 ml of ACCM‐D for 24 h and then plated onto ACCM‐agarose medium containing chloramphenicol (5 μg/ml) for 6 days. Single colonies were subsequently picked, resuspended in 1 ml of chloramphenicol‐supplemented ACCM‐D in 24‐well plates, and cultured for another 6 days. To identify the insertion site of the transposon in the genome, a semi‐random, two‐step PCR (ST‐PCR) reactions^62^ with nested primers within the transposon was employed. For each 20‐μl ST‐PCR reaction^62^, 1 μl of the genomic DNA was used. The first round of amplification used primer 1: 5′‐GGGGGAAACGCCTGGTATC‐3′ and a pool of random oligonucleotide primers 2: 5′‐GCCGGAGCTCTGCAGAATTC NNNNNNNN‐3′. The product of this reaction was used as the template for the second round of amplification, using primer 3: 5′‐GTCGGGTTTCGCCACCTC and primer ARB2: 5′‐GGCCAGGCCTGCAGATGATG‐3′. Product of the second PCR reaction was used to determine the site of insertion by sequencing using primer 3: 5′‐TCGATTTTTGTGATGCTCGTC‐3′^38^.

To compare the growth of bacterial strains in synthetic medium, cultures of the wild‐type or the *cubL1*::Tn strain were diluted 1:100 in ACCM‐D media. Samples were collected at 24 h intervals for 7 days. Genomic DNA was extracted from the samples using the Illustra Bacteria GenomicPrep Mini Prep Kit (GE Healthcare). This DNA was then used to quantify genomic equivalents via *com1*‐specific quantitative PCR (qPCR)^63^.

To determine intracellular growth, THP‐1 cells were seeded at a density of 2 × 10^4^/well in 24‐well plates for 24 h with PMA at 100 nM, and then infected with *C. burnetii* strains, axenically grown to the stationary phase. The concentration of the bacterial cultures was determined by qPCR using *com1*‐specific primers^64^, and the bacterial cell density was adjusted with DMEM containing 2% FBS to achieve an MOI of 100. After incubating the infected samples for 4 h, we washed them once with PBS and then incubated them in fresh DMEM containing 2% FBS. We designated this time as Day 0 and collected samples to determine the inoculum. We then prepared cell lysates from samples collected at 1‐day intervals over 7 days. Genomic DNA was extracted from these samples using the Illustra Bacteria GenomicPrep Mini Prep Kit (GE Healthcare) and used to quantify genomic equivalents via *com1*‐specific quantitative PCR (qPCR).

### Detection of CubL1‐mediated eIF3g ubiquitination in cells infected by *C. burnetii*

Lentiviral particles that allow the expression of Flag‐eIF3g were prepared as described earlier^65^ and were used to infect THP‐1 cells. Transduced cells were in medium containing 2 μg/ml puromycin^66^, clones were obtained by serial dilutions, and one line (THP‐1‐Flag‐eIF3g) expressing Flag‐eIF3g was isolated by immunoblotting with Flag‐specific antibody.

Cells of the THP‐1‐Flag‐eIF3g line were seeded at a density of 10^6^/ml in 10 cm plates in medium containing 100 nM PMA for 24 h prior to being infected with the appropriate *C. burnetii* strains, axenically grown to the stationary phase. The concentration of the bacteria in cultures was determined by qPCR using *com1*‐specific primers^64^, and the bacterial cell density was adjusted with RPMI 1640 containing 2% FBS to achieve an MOI of 200. After incubation for 4 h, infected samples were washed once with PBS and incubated with fresh DMEM containing 2% FBS. Cells were collected and lysed with RIPA buffer 3 days post infection, and cleared lysates were subjected to immunoprecipitation with agarose beads coated with Flag‐specific antibody by incubating on a rotatory shaker for 4 h at 4°C. Beads were washed three times with pre‐chilled RIPA buffer, and bound proteins separated by SDS‐PAGE were detected by CBB staining. Protein bands corresponding to Ub‐Flag‐eIF3g were excised and digested with trypsin as described^67^, and the ubiquitinated peptide was detected by LC‐MS analysis.

### Protein expression and purification

To produce GST‐CubL1, GST‐eIF3g_1–230_, and GST‐SdeA_1–200_, the corresponding region of the gene of interest amplified by PCR from appropriate templates with specific primer pairs (Table S2) was inserted into pGEX‐6P‐1 (Qiagen) as *Bam*HI and *Sal*I fragments. His_6_‐eIF3g and His_6_‐eIF3g‐N (1–230) were purified using plasmids derived from pET‐28a that carry the corresponding protein fragment. *E. coli* strains derived from BL21(DE3) with the plasmids were used for protein expression. Briefly, bacterial cultures at mid‐exponential phase were induced with 0.2 mM IPTG for 16 h at 20°C. Bacteria were lysed using PBS buffer containing 1% Triton. GST‐ and His_6_‐tagged proteins were purified by affinity chromatography using Glutathione‐Sepharose and Ni^2+^‐NTA beads, respectively. All purified proteins had more than 95% purity as assessed by SDS‐PAGE and CBB staining. Proteins were dialyzed and saved at −80°C in a storage buffer (20 mM Tris‐HCl pH 8.0, 200 mM NaCl, 1 mM DTT, and 10% glycerol).

To produce ubiquitinated GST‐eIF3g, ubiquitination assays were performed at 37°C for 3 h in a reaction buffer containing 50 mM Tris‐HCl (pH 7.5), 2 mM ATP, 5 mM Mg^2+^, and 1 mM DTT. Each 10‐ml reaction contains 1 mg His‐Ub, 5 μg E1, 5 μg UbcH5C, 1 mg CubL1, and 1 mg GST‐eIF3g; ubiquitinated GST‐eIF3g protein was purified by affinity chromatography first using Ni^2+^‐NTA beads, and then using Glutathione‐Sepharose column.

For expression in mammalian cells, the testing genes were inserted into pCMV4Flag^68^ to give pFlagCubL1. The cDNA of full‐length or fragments of the *eIF3g* were inserted into the pCMV4Flag^69^ or pEGFPC1(Clontech). Caspase‐3 and its mutant were constructed. Various point mutants of *CubL1* or *eIF3g* were generated by PCR using primers with desired mutations (Table S2).

To purify protein from mammalian cells, HEK293T cells were transfected with GFP‐ or Flag‐tagged proteins for 12 h. Lysates of transfected cells were subjected to immunoprecipitation with agarose beads coated with an antibody specific for GFP or Flag. After incubation at 4°C for 4 h, bound proteins were eluted with Flag peptides.

### Biacore analysis

The affinity of CubL1 for Ub or UbcH5C was analyzed using a Biacore X100 device (Biacore Inc.) according to the manufacturer's protocol. A CM5 chip (General Electric Company) was used to immobilize the CubL1 protein. Various concentrations of Ub or UbcH5C were injected, and all steps were performed in HBS‐EP buffer (GE). Data analysis was carried out using Biacore Evaluation Software version 3.1 and the 1:1 Langmuir binding model.

### Identification of the cellular targets of CubL1

HEK293T cells grown to 70% confluence in 100 mm tissue culture dishes were transfected to express the Flag‐CubL1‐Ub with 10 μg DNA using Lipofectamine 3000 for 18 h. Cells from two plates were collected in a 15‐ml conical tube by centrifugation at 1100*g* for 5 min. Cells resuspended in 8‐ml NP40 lysis buffer were incubated on ice for 10 min. Cleared lysates were obtained by centrifugation at 13,000*g* for 10 min, transferred to a new test tube, and 50 μl Flag‐antibody beads were added prior to incubating on a rotatory shaker for 12 h at 4°C. Beads were washed three times, each with 1‐ml PBS. Proteins were solubilized with 75‐μl Laemmli buffer on the beads, and proteins were denatured by boiling the samples at 95°C for 5 min. Samples were separated by SDS‐PAGE, and proteins were processed by in‐gel tryptic digestion and analyzed by LC‐MS/MS.

### Biochemical ubiquitination assays

Ubiquitination assays were performed at 37°C in 50 μl reactions in a buffer that contains 50 mM Tris‐HCl (pH 8.0), 50 mM NaCl, 10 mM MgCl_2_, 0.1 mM DTT, 5 mM ATP, 100 nM E1, 200 nM E2, 0.5 μM CubL1, and 100 μM Ub. For dropout experiments, the indicated reactants were withdrawn. Reactions were allowed to proceed for 2 h prior to being terminated by adding 12.5 μl 5× Laemmli buffer containing 100 mM DTT. Proteins resolved by SDS‐PAGE were detected by CBB staining or by immunoblotting.

### Immunoprecipitation

Transfected cells were collected and lysed with the RIPA buffer at 16–18 h post transfection. Cleared lysates obtained by centrifugation at 13,000*g* for 10 min were subjected to immunoprecipitation using agarose beads coated with Flag‐specific antibody (Sigma) by incubating on a rotatory shaker at 4°C for 4 h. Beads were washed three times with pre‐chilled RIPA buffer, and samples solubilized with the Laemmli buffer were resolved by SDS–PAGE. Proteins transferred onto nitrocellulose membranes were detected by immunoblotting with appropriate antibodies.

### Antibodies and immunoblotting

Antibodies specific for CubL1 were produced using a standard protocol^70^, ^71^. Briefly, 1 mg of GST‐CubL1 was emulsified with an equal volume of complete Freund's adjuvant and was injected intracutaneously into mice four times a month at a 7‐day interval. Sera of the immunized mice that contain CubL1‐specific antibodies were used for affinity purification of IgG, according to an established protocol^72^.

For immunoblotting, cells were lysed by the RIPA buffer (50 mM Tris (pH 7.4), 150 mM NaCl, 1% sodium‐deoxycholate, and 1% NP‐40) supplemented with a protease inhibitor cocktail (Roche Molecular Biochemicals). Protein concentration was determined using the BCA Protein Assay Kit (Thermo Scientific Pierce) prior to being denatured in the Laemmli buffer by boiling for 5 min. Samples were then resolved by SDS‐PAGE, after which the proteins were transferred onto nitrocellulose membranes (Millipore). After blocking in 5% nonfat milk in TBST (150 mM NaCl, 20 mM Tris‐HCl (pH 7.4, 0.1% Tween‐20), the membranes were incubated with primary antibodies at the following dilutions: anti‐Flag (Cat# F1804, 1:3000; Sigma), anti‐GST (1:2000), anti‐GAPDH (Cat# 5174, 1:5000; Cell signaling), anti‐CubL1 (1:1000), and anti‐Ub (1:200). Washed membranes were incubated in the same buffer containing IRDye 680‐ or 800‐conjugated secondary antibodies (1:10,000; Abcam), and after another three times of washes, signals were detected using an Odyssey infrared imaging system (Li‐Cor's Biosciences Lincoln). Signal quantification was carried out using the same system following the instructions provided by the manufacturer.

### Molecular modeling

The structure of CubL1 or eIF3g was predicted by the AlphaFold system^33^. The ubiquitin structure (PDB:1UBQ) was used for modeling. Standard protein‐ligand docking was performed using AutoDock4 to generate the initial structure for molecular simulation with the Gromacs 4.5.5 package.

### DNase activity assays

Genomic DNA isolated from mammalian cells using the TIANamp Genomic DNA kit was used as the substrate in the DNase activity assay. Two micrograms of DNA was incubated with purified 3 μg eIF3g‐N (1–230), 3 μg Ub‐eIF3g‐N (1–230), or 50 μl of reaction buffer (20 mM NaCl, 10 mM Tris‐HCl, 2.5 mM MgCl_2_) for 1 h at 37°C. To remove the Ub moiety from Ub‐eIF3g‐N, 1 μg SdeA_1–200_ was added to the reactions. The integrity of DNA was analyzed by ethidium bromide staining after electrophoresis in 1.0% agarose gels. Images were acquired using a Tanon mini space series gel image analysis system.

### Flow cytometry and TUNEL staining

THP‐1 cells seeded at a density of 2 × 10^4^/well in 24‐well plates for 24 h were infected with the appropriate *C. burnetii* strains, axenically grown to the stationary phase. The concentration of the bacterial cultures was determined by qPCR using *com1*‐specific primers^9^, and the bacterial cell density was adjusted with DMEM containing 2% FBS to achieve an MOI of 200. After incubation for 4 h, infected samples were washed once with PBS and incubated with fresh DMEM containing 2% FBS. Infected cells were collected at 1‐day intervals for 6 days. For samples used for flow cytometry analysis, infected samples were first treated with STS for 4 h, followed by staining with Annexin V‐FITC. Cell sorting analysis was performed using a Beckman CytoFLEX set at 515–565 nm excitation wavelength and a 450–500 nm emission wavelength.

For TUNEL assays, THP‐1 cells infected at the indicated time points were fixed with 3.7% (vol/vol) formaldehyde for 15 min at room temperature. After permeabilization with 0.1% Triton X‐100 for 10 min, samples were blocked with 4% goat serum for 30 min at 37°C, and bacteria were stained with anti‐*C. burnetii* sera at a dilution of 1:1000 for 2 h, and DNA was stained with a one‐step TUNEL cell apoptosis detection kit. To stain transfected cells, samples treated with STS for 4 h were similarly processed. The rates of apoptotic cells and image acquisition were obtained using an Olympus IX‐83 microscope.

### Detection of protein synthesis in cells

Protein synthesis was detected using the Click‐iT kit following the manufacturer's instructions. Briefly, 5 × 10^4^ HeLa cells in 24‐well plates were transfected to express GFP, GFP‐SidI, or GFP‐CubL1 for 16 h. Cells were washed two times with PBS and were incubated with l‐methionine‐free medium containing 50 μM HPG for 45 min. After washes three times with PBS containing 3% BSA, cells were fixed with 4% polyoxymethylene for 15 min. Cells were washed three times with 3% BSA in PBS prior to being permeabilized with 0.5% Triton for 10 min. A 100‐μl Click‐iT reaction cocktail was added to the samples and incubated in the dark for 30 min. The reaction medium was removed, and cells were washed once with 100 μl rinse buffer. Finally, samples were incubated in 100 μl 1×HCS NuclearMask Blue Stain to stain DNA for 30 min. Samples were inspected using an IX‐83 Olympus fluorescence microscope.

### LC‐MS/MS analysis

Protein bands were digested in‐gel with trypsin as previously described^67^. In‐gel digestion was performed as previously described^67^. LC‐ESI‐MS/MS analysis was carried out using an EASY‐nLC 1200 system coupled to an Orbitrap Exploris 240 mass spectrometer (Thermo Scientific), with chromatographic conditions and acquisition parameters as detailed^67^. Mobile phase composition, gradient program, and mass spectrometer settings (including resolution, AGC targets, ion injection times, and dynamic exclusion) followed a standard nanoflow proteomics workflow^67^. Raw data processing was performed using MaxQuant (version 1.6.3.3) against the *C. burnetii* (UP000002671) or *Homo sapiens* (UP000005640) database. Search parameters (peptide tolerance of 10 ppm, MS/MS tolerance of 0.02 Da, carbamidomethyl fixed, oxidation variable, up to two missed cleavages) were set according to standard MaxQuant settings for Orbitrap data. For ubiquitination site identification, the diglycine remnant on lysine residues was included as a variable modification following established protocols^67^, ^73^.

## AUTHOR CONTRIBUTIONS

**Yong Zhang**: Data curation; formal analysis; funding acquisition; methodology; writing—original draft; writing—review and editing. **Jiaqi Fu**: Formal analysis; funding acquisition. **Zhao‐Qing Luo**: Project administration; writing—original draft; writing—review and editing.

## ETHICS STATEMENT

All procedures involving animals​ for antibody production were conducted in strict accordance with institutional and national guidelines for the care and use of laboratory animals and approved by the Institutional Animal Care and Use Committee (IACUC) of Jilin University (Approval No. 20200669). No human research was involved in this study.

## CONFLICT OF INTERESTS

The authors declare no conflict of interests.

## Supporting information

Supporting File 1.

Supporting File 2.

Supporting File 3.

## Data Availability

Data supporting the findings of this study are included in the manuscript. Requests for additional materials should be directed to the corresponding authors.
