# Supplementary material for: Coxiella burnetii inhibits host cell death by ubiquitination‐induced inactivation of an apoptosis nuclease
Source: mLife. 2026 Aug 27;5(4):432–46. doi: 10.1002/mlf2.70093 (PMC13519585; doi:10.1002/mlf2.70093)
Supplement: Supplementary file 1 [file MLF2-5-432-s001.docx]

| **Protein** | **Peptides** | **PSMs** |
| --- | --- | --- |
| UPF0422 protein CBU_0937 OS=Coxiella burnetii (strain RSA 493 / Nine Mile phase I) OX=227377 GN=CBU_0937 PE=3 SV=1 | 20 | 211 |
| Uncharacterized protein OS=Coxiella burnetii (strain RSA 493 / Nine Mile phase I) OX=227377 GN=CBU_0021 PE=4 SV=1 | 36 | 209 |
| Uncharacterized protein OS=Coxiella burnetii (strain RSA 493 / Nine Mile phase I) OX=227377 GN=CBU_2013 PE=4 SV=1 | 18 | 114 |
| Uncharacterized protein OS=Coxiella burnetii (strain RSA 493 / Nine Mile phase I) OX=227377 GN=CBU_1665 PE=4 SV=1 | 3 | 57 |
| Uncharacterized protein CBU_0041 OS=Coxiella burnetii (strain RSA 493 / Nine Mile phase I) OX=227377 GN=CBU_0041 PE=4 SV=1 | 3 | 43 |
| Uncharacterized protein OS=Coxiella burnetii (strain RSA 493 / Nine Mile phase I) OX=227377 GN=CBU_1752 PE=4 SV=1 | 15 | 41 |
| Hypothetical membrane spanning protein OS=Coxiella burnetii (strain RSA 493 / Nine Mile phase I) OX=227377 GN=CBU_1863 PE=4 SV=1 | 12 | 40 |
| Uncharacterized protein OS=Coxiella burnetii (strain RSA 493 / Nine Mile phase I) OX=227377 GN=CBU_1686 PE=4 SV=1 | 10 | 35 |
| 17 kDa common-antigen OS=Coxiella burnetii (strain RSA 493 / Nine Mile phase I) OX=227377 GN=CBU_1425 PE=4 SV=1 | 5 | 27 |
| Uncharacterized protein OS=Coxiella burnetii (strain RSA 493 / Nine Mile phase I) OX=227377 GN=CBUA0013 PE=4 SV=1 | 4 | 19 |
| Uncharacterized protein OS=Coxiella burnetii (strain RSA 493 / Nine Mile phase I) OX=227377 GN=CBU_1751 PE=4 SV=1 | 6 | 18 |
| Uncharacterized protein CBU_1754 OS=Coxiella burnetii (strain RSA 493 / Nine Mile phase I) OX=227377 GN=CBU_1754 PE=1 SV=1 | 3 | 17 |
| Uncharacterized protein OS=Coxiella burnetii (strain RSA 493 / Nine Mile phase I) OX=227377 GN=CBU_1409 PE=4 SV=1 | 2 | 17 |
| Putative ankyrin repeat protein CBU_0781 OS=Coxiella burnetii (strain RSA 493 / Nine Mile phase I) OX=227377 GN=CBU_0781 PE=4 SV=1 | 1 | 15 |
| Fic family protein OS=Coxiella burnetii (strain RSA 493 / Nine Mile phase I) OX=227377 GN=CBU_2078 PE=4 SV=1 | 4 | 15 |
| Tetratricopeptide repeat family protein OS=Coxiella burnetii (strain RSA 493 / Nine Mile phase I) OX=227377 GN=CBU_1457 PE=4 SV=1 | 5 | 13 |
| Uncharacterized protein OS=Coxiella burnetii (strain RSA 493 / Nine Mile phase I) OX=227377 GN=CBUA0023 PE=4 SV=1 | 4 | 12 |
| Probable transcriptional regulatory protein CBU_1566 OS=Coxiella burnetii (strain RSA 493 / Nine Mile phase I) OX=227377 GN=CBU_1566 PE=1 SV=1 | 5 | 11 |
| Hypothetical cytosolic protein OS=Coxiella burnetii (strain RSA 493 / Nine Mile phase I) OX=227377 GN=CBU_1569 PE=4 SV=1 | 5 | 10 |
| Uncharacterized protein OS=Coxiella burnetii (strain RSA 493 / Nine Mile phase I) OX=227377 GN=CBU_2007 PE=4 SV=1 | 5 | 9 |
| Uncharacterized protein OS=Coxiella burnetii (strain RSA 493 / Nine Mile phase I) OX=227377 GN=CBU_1685 PE=4 SV=1 | 3 | 7 |
| Uncharacterized protein OS=Coxiella burnetii (strain RSA 493 / Nine Mile phase I) OX=227377 GN=CBU_1794 PE=4 SV=2 | 6 | 7 |
| Uncharacterized protein OS=Coxiella burnetii (strain RSA 493 / Nine Mile phase I) OX=227377 GN=CBU_1543 PE=4 SV=1 | 2 | 5 |
| Hypothetical cytosolic protein OS=Coxiella burnetii (strain RSA 493 / Nine Mile phase I) OX=227377 GN=CBU_1387 PE=4 SV=1 | 2 | 5 |
| Hypothetical membrane associated protein OS=Coxiella burnetii (strain RSA 493 / Nine Mile phase I) OX=227377 GN=CBU_0534 PE=4 SV=1 | 2 | 5 |
| Hypothetical membrane spanning protein OS=Coxiella burnetii (strain RSA 493 / Nine Mile phase I) OX=227377 GN=CBU_1576 PE=4 SV=1 | 2 | 4 |
| Uncharacterized protein OS=Coxiella burnetii (strain RSA 493 / Nine Mile phase I) OX=227377 GN=CBU_0665 PE=4 SV=1 | 3 | 4 |
| Uncharacterized protein OS=Coxiella burnetii (strain RSA 493 / Nine Mile phase I) OX=227377 GN=CBU_0295 PE=4 SV=1 | 3 | 4 |
| Uncharacterized protein OS=Coxiella burnetii (strain RSA 493 / Nine Mile phase I) OX=227377 GN=CBU_1724 PE=4 SV=1 | 4 | 4 |
| Uncharacterized protein OS=Coxiella burnetii (strain RSA 493 / Nine Mile phase I) OX=227377 GN=CBU_1493 PE=4 SV=2 | 4 | 4 |
| Ankyrin repeat protein OS=Coxiella burnetii (strain RSA 493 / Nine Mile phase I) OX=227377 GN=CBU_0072 PE=4 SV=1 | 2 | 4 |
| Hypothetical membrane spanning protein OS=Coxiella burnetii (strain RSA 493 / Nine Mile phase I) OX=227377 GN=CBU_0077 PE=4 SV=1 | 3 | 3 |
| GatB/YqeY domain protein OS=Coxiella burnetii (strain RSA 493 / Nine Mile phase I) OX=227377 GN=CBU_1594 PE=4 SV=1 | 3 | 3 |
| Ankyrin repeat protein OS=Coxiella burnetii (strain RSA 493 / Nine Mile phase I) OX=227377 GN=CBU_0447 PE=4 SV=1 | 2 | 3 |
| Hypothetical cytosolic protein OS=Coxiella burnetii (strain RSA 493 / Nine Mile phase I) OX=227377 GN=CBU_1676 PE=4 SV=1 | 3 | 3 |
| Uncharacterized protein OS=Coxiella burnetii (strain RSA 493 / Nine Mile phase I) OX=227377 GN=CBUA0020 PE=4 SV=1 | 2 | 3 |
| Hypothetical membrane spanning protein OS=Coxiella burnetii (strain RSA 493 / Nine Mile phase I) OX=227377 GN=CBU_0410 PE=4 SV=1 | 3 | 3 |
| Uncharacterized protein OS=Coxiella burnetii (strain RSA 493 / Nine Mile phase I) OX=227377 GN=CBU_0794 PE=4 SV=1 | 3 | 3 |
| Uncharacterized protein CBU_1818 OS=Coxiella burnetii (strain RSA 493 / Nine Mile phase I) OX=227377 GN=CBU_1818 PE=4 SV=1 | 1 | 2 |
| Uncharacterized protein OS=Coxiella burnetii (strain RSA 493 / Nine Mile phase I) OX=227377 GN=CBU_1790 PE=4 SV=1 | 1 | 2 |
| Uncharacterized protein OS=Coxiella burnetii (strain RSA 493 / Nine Mile phase I) OX=227377 GN=CBU_1639 PE=4 SV=1 | 1 | 2 |
| Uncharacterized protein OS=Coxiella burnetii (strain RSA 493 / Nine Mile phase I) OX=227377 GN=CBU_0425 PE=4 SV=1 | 1 | 2 |
| Hypothetical membrane spanning protein OS=Coxiella burnetii (strain RSA 493 / Nine Mile phase I) OX=227377 GN=CBU_1556 PE=4 SV=1 | 2 | 2 |
| Uncharacterized protein OS=Coxiella burnetii (strain RSA 493 / Nine Mile phase I) OX=227377 GN=CBU_0626 PE=4 SV=1 | 2 | 2 |
| Uncharacterized protein OS=Coxiella burnetii (strain RSA 493 / Nine Mile phase I) OX=227377 GN=CBU_0388 PE=4 SV=1 | 2 | 2 |
| Uncharacterized protein OS=Coxiella burnetii (strain RSA 493 / Nine Mile phase I) OX=227377 GN=CBU_1063 PE=4 SV=1 | 2 | 2 |
| Hypothetical cytosolic protein OS=Coxiella burnetii (strain RSA 493 / Nine Mile phase I) OX=227377 GN=CBU_1198 PE=4 SV=1 | 2 | 2 |
| Ankyrin repeat protein OS=Coxiella burnetii (strain RSA 493 / Nine Mile phase I) OX=227377 GN=CBU_1213 PE=4 SV=1 | 2 | 2 |
| Hypothetical membrane spanning protein OS=Coxiella burnetii (strain RSA 493 / Nine Mile phase I) OX=227377 GN=CBU_1217 PE=4 SV=1 | 2 | 2 |
| Uncharacterized protein CBU_1819 OS=Coxiella burnetii (strain RSA 493 / Nine Mile phase I) OX=227377 GN=CBU_1819 PE=4 SV=1 | 2 | 2 |
| Gluconolactonase OS=Coxiella burnetii (strain RSA 493 / Nine Mile phase I) OX=227377 GN=CBU_1789 PE=4 SV=1 | 1 | 1 |
| Hypothetical membrane spanning protein OS=Coxiella burnetii (strain RSA 493 / Nine Mile phase I) OX=227377 GN=CBU_0635 PE=4 SV=1 | 1 | 1 |
| Uncharacterized protein OS=Coxiella burnetii (strain RSA 493 / Nine Mile phase I) OX=227377 GN=CBU_1607 PE=4 SV=1 | 1 | 1 |
| Uncharacterized protein CBUA0006 OS=Coxiella burnetii (strain RSA 493 / Nine Mile phase I) OX=227377 GN=CBUA0006 PE=4 SV=1 | 1 | 1 |
| Hypothetical cytosolic protein OS=Coxiella burnetii (strain RSA 493 / Nine Mile phase I) OX=227377 GN=CBU_0881 PE=4 SV=1 | 1 | 1 |
| Serine/threonine protein kinase domain protein OS=Coxiella burnetii (strain RSA 493 / Nine Mile phase I) OX=227377 GN=CBU_0175 PE=4 SV=1 | 1 | 1 |
| Hypothetical cytosolic protein OS=Coxiella burnetii (strain RSA 493 / Nine Mile phase I) OX=227377 GN=CBU_1314 PE=4 SV=2 | 1 | 1 |
| Uncharacterized protein OS=Coxiella burnetii (strain RSA 493 / Nine Mile phase I) OX=227377 GN=CBU_1178 PE=4 SV=1 | 1 | 1 |
| Hypothetical cytosolic protein OS=Coxiella burnetii (strain RSA 493 / Nine Mile phase I) OX=227377 GN=CBU_2016 PE=4 SV=2 | 1 | 1 |
