# Supplementary material for: Coxiella burnetii inhibits host cell death by ubiquitination‐induced inactivation of an apoptosis nuclease
Source: mLife. 2026 Aug 27;5(4):432–46. doi: 10.1002/mlf2.70093 (PMC13519585; doi:10.1002/mlf2.70093)
Supplement: Supplementary file 3 [file MLF2-5-432-s003.docx]

**Bacterial Strains used in this study**

| **Designation** | **SOURCE** | **IDENTIFIER** |
| --- | --- | --- |
| C. burnetii Nine Mile phase II (RSA493) |  | N/A |
| *icmD::Tn* | This paper | N/A |
| *cubL1::Tn* | This paper | N/A |
| *cubL1::Tn*(pCubL1) | This paper | N/A |
| *cubL1::Tn*(pCubL1_3R_) | This paper | N/A |

**Plasmids used in this study**

| **name** | **source** |
| --- | --- |
| pJB-Kan-P1169-3xFLAG | (Beare et al, 2012) |
| pJB-Flag-TEM1-EliC1 | This paper |
| pEGFPC1-EliC1 | This paper |
| pCMV4xFlag | (Li and Qiu et al, 2020) |
| pET28a | Invitrogen |
| pET28a-EliC1 | This paper |
| pGEX6p-1-EliC1 | This paper |
| pGEX6p-1-EliC1_C22A_ | This paper |
| pGEX6p-1-EliC1_C69A_ | This paper |
| pGEX6p-1-EliC1_C220A_ | This paper |
| pGEX6p-1-EliC1_C232A_ | This paper |
| pGEX6p-1-EliC1_C250A_ | This paper |
| pGEX6p-1-EliC1_C314A_ | This paper |
| pGEX6p-1-EliC1_C372A_ | This paper |
| pGEX6p-1-EliC1_E164R_ | This paper |
| pGEX6p-1-EliC1_E166R_ | This paper |
| pGEX6p-1-EliC1_E164R/E166R_ | This paper |
| pGEX6p-1-EliC1_Q196R_ | This paper |
| pGEX6p-1-EliC1_E228R_ | This paper |
| pGEX6p-1-EliC1_Q225R/E228R_ | This paper |
| pGEX6p-1-EliC1_Q196R/Q225R/E228R_ | This paper |
| pET28a-Uniquitin | This paper |
| pET28a-Uniquitin(K6A) | This paper |
| pET28a-Uniquitin(K11A) | This paper |
| pET28a-Uniquitin(K27A) | This paper |
| pET28a-Uniquitin(K29A) | This paper |
| pET28a-Uniquitin(K33A) | This paper |
| pET28a-Uniquitin(K48A) | This paper |
| pET28a-Uniquitin(K63A) | This paper |
| pET28a-Uniquitin(KO) | This paper |
| pET28a-Uniquitin(G75A,G76A) | This paper |
| pET28a-UbcH5c | This paper |
| pET28a-UbcH7 | This paper |
| pET28a-E1 | This paper |
| pET28a-eIF3g | This paper |
| pET28a-eIF3g(K78R) | This paper |
| pET28a-eIF3g1-230 | This paper |
| pET28a-eIF3g1-230 (K78R) | This paper |
| pCMV | Invitrogen |
| pJB-Flag-TEM1-EliC1 | This paper |
| pCMV-Flag-EliC1 | This paper |
| pCMV-Flag-eIF3g | This paper |
| pCMV-Flag-eIF3g(K78R) | This paper |
| pCMV-Flag-eIF3g1-230 | This paper |
| pCMV-Flag-eIF3g1-230 (K78R) | This paper |
| pJB-Flag-EliC1 | This paper |
| pCMV-Flag-Cbu1576 | This paper |
| pCMV-Flag-Cbu1794 | This paper |
| pCMV-Flag-Cbu1724 | This paper |
| pCMV-Flag-Cbu1556 | This paper |
| pCMV-Flag-Cbu1569 | This paper |
| pQE30-SdeA_(1-200)_ | This paper |

**Reference:**

[1] Beare PA. Genetic manipulation of Coxiella burnetii. Advances in experimental medicine and biology 2012;984:249-71.

[2] Li G, Liu H, Luo ZQ, Qiu J. Modulation of phagosome phosphoinositide dynamics by a Legionella phosphoinositide 3-kinase. EMBO reports 2021;22:e51163.

**Primers used in this study**

| Primer name | Sequence (Restriction enzyme sites are underlined) | Note |
| --- | --- | --- |
| EliC1-F WT | CGCGGATCCTTGAGGTACAAGCGACACATG | EliC1 5’ BamH I |
| EliC1-R WT | CTAGTCGACTTAAAAAGTAAAGGATTGTTTAGAG | EliC1 3’ Sal I |
| EliC1-F | CGCGGATCCATGAGATATAAGAGGCACATGGA | EliC1 5’ BamH I |
| EliC1-R | CTAGTCGACGTGGTGGTGGTGGTGGTGAAAGGTAAAGCTCTGCTTGC | EliC1 3’ Sal I |
| EliC1_C22A_-F | CTCACGACTTTTGCGAAAGGCCTCAATAGCGAAGTCAATG | EliC1 mutant C22A |
| EliC1_C22A_-R | CATTGACTTCGCTATTGAGGCCTTTCGCAAAAGTCGTGAG |  |
| EliC1_C69A_-F | GGTAATGTAGTTGAAGTAATTCACAGCGTCTTCATGCGGTTTATCATTGAAA | EliC1 mutant C69A |
| EliC1_C69A_-R | TTTCAATGATAAACCGCATGAAGACGCTGTGAATTACTTCAACTACATTACC |  |
| EliC1_C220A_-F | GATTTGATACTTATTACCGGCCACGGCGAACAGACGCGC | EliC1 mutant C220A |
| EliC1_C220A_-R | GCGCGTCTGTTCGCCGTGGCCGGTAATAAGTATCAAATC |  |
| EliC1_C232A_-F | GAACAGCACCGCTTCATAAGCGCGCTTGGCTTCTTTGATT | EliC1 mutant C232A |
| EliC1_C232A_-R | AATCAAAGAAGCCAAGCGCGCTTATGAAGCGGTGCTGTTC |  |
| EliC1_C250A_-F | TTCTCATGCTCCAGAAAGGCGGCAAAGCGGTAGATTGC | EliC1 mutant C250A |
| EliC1_C250A_-R | GCAATCTACCGCTTTGCCGCCTTTCTGGAGCATGAGAA |  |
| EliC1_C314A_-F | TTTTCGATCGCCTCAATGGCATATTCGATCGCCTTGCCG | EliC1 mutant C314A |
| EliC1_C314A_-R | CGGCAAGGCGATCGAATATGCCATTGAGGCGATCGAAAA |  |
| EliC1_C372A_-F | GTAGTTTTTCAGGCTGTTGGCGTGCTTGATCGCCAATGGC | EliC1 mutant C372A |
| EliC1_C372A_-R | GCCATTGGCGATCAAGCACGCCAACAGCCTGAAAAACTAC |  |
| EliC1_E164R_-F | TTCTCCAGCTCTTCTTTCCTGAGAGAGTGGTTGGCAGC | EliC1 mutant E164R |
| EliC1_E164R_-R | GCTGCCAACCACTCTCTCAGGAAAGAAGAGCTGGAGAA |  |
| EliC1_E166R_-F | CGTTCTCCAGCTCTCTTTTCTCGAGAGAGTGGTTGGCAG | EliC1 mutant E166R |
| EliC1_E166R_-R | CTGCCAACCACTCTCTCGAGAAAAGAGAGCTGGAGAACG |  |
| EliC1_E164R/E166R_-F | GTCTTAGCGTTCTCCAGCTCTCTTTTCCTGAGAGAGTGGTTGGCAGCCAA | EliC1 mutant E164R/E166R |
| EliC1_E164R/E166R_-R | TTGGCTGCCAACCACTCTCTCAGGAAAAGAGAGCTGGAGAACGCTAAGAC |  |
| EliC1_Q196R_-F | ACAGTGGCGTAAGATTCCGACGTTGATACAGGAAG | EliC1 mutant Q196R |
| EliC1_Q196R_-R | CTTCCTGTATCAACGTCGGAATCTTACGCCACTGT |  |
| EliC1_Q225R_-F | GCTTGGCTTCTTTGATTCTATACTTATTACCGCACACGGCG | EliC1 mutant Q225R |
| EliC1_Q225R_-R | CGCCGTGTGCGGTAATAAGTATAGAATCAAAGAAGCCAAGC |  |
| EliC1_E228R_-F | TAACAGCGCTTGGCTCTTTTGATTTGATACTTATTACCGCACACGG | EliC1 mutant E228R |
| EliC1_E228R_-R | CCGTGTGCGGTAATAAGTATCAAATCAAAAGAGCCAAGCGCTGTTA |  |
| EliC1_Q225R/E228R_-F | TAACAGCGCTTGGCTCTTTTGATTCTATACTTATTACCGCACACGGCGAACAGAC | EliC1 mutant Q225R/E228R |
| EliC1_Q225R/E228R_-R | GTCTGTTCGCCGTGTGCGGTAATAAGTATAGAATCAAAAGAGCCAAGCGCTGTTA |  |
| eIF3g-F | CTGAGATCTATGCCTACTGGAGACTTTGATTC | eIF3g 5’ BglⅡ |
| eIF3g-R | CTGGTCGACTTAGTTGGTGGACGGCTTGGC | eIF3g 3’ Sal I |
| HSP90α-F | CTGGGATCCATGCCTGAGGAAACCCA | HSP90α 5’ BamH I |
| HSP90α-R | CTGGTCGACTTAGTCTACTTCTTCCATGCGT | HSP90α 3’ Sal I |
| MYH9-F | CTGGGATCCATGGCACAGCAAGCTGCC | MYH9 5’ BamH I |
| MYH9-R | CTGGTCGACTTATTCGGCAGGTTTGGCCT | MYH9 3’ Sal I |
| P1169 Promoter-F | CTGCTCGAGATGGCTTCGTTTCGCAGCG | P1169 Promoter 5’ Xho I |
| P1169 Promoter-R | CTGGAGCTCCCTCCTTCATGAAGGATTAA | P1169 Promoter 3’ Sac I |
| TEM1-F | GTCGTCGACCCAGAAACGCTGGTGAAAGT | TEM1 5’ Sal I |
| TEM1-R | CTTGGATCCTTACCAATGCTTAATCAGTGAG | TEM1 3’ BamH I |
| Com1-F | AAAACCTCCGCGTTGTCTTCA | Quantify the amount of C. burnetii genomic |
| Com1-R | GCTAATGATACTTTGGCAGCGTATTG |  |
| Com1-probe | AGAACTGCCCATTTTTGGCGGCCA | 5`6-FAM，3`BHQ1 |
| CBU1794-F | CTGGGATCCATGGAGGCTGATATCATG | CBU1794 5’ BamH I |
| CBU1794-R | CTGGTCGACCTATCGATGACTCGTTAA | CBU1794 3’ Sal I |
| CBU1576-F | CTGGGATCCATGCGTCCAGAACATAAA | CBU1576 5’ BamH I |
| CBU1576-R | CTGCTCGAGCTAATGTTTCATCGAATA | CBU1576 3’ Xho I |
| CBU1724-F | CTGGGATCCATGACTCGGCAGATCAAT | CBU1724 5’ BamH I |
| CBU1724-R | CTGGTCGACCTATTTCAGGCCGGGGGG | CBU1724 3’ Sal I |
| CBU1556-F | CTGGGATCCATGCCTTCTGATAGAAAC | CBU1556 5’ BamH I |
| CBU1556-R | CTGGTCGACCTAAACGGGAGCATAATT | CBU1556 3’ Sal I |
| CBU1569-F | CTGGGATCCATGCCTATTACCAGCTTA | CBU1569 5’ BamH I |
| CBU1569-R | CTGGTCGACTTAATTGTTATTTCGAGG | CBU1569 3’ Sal I |
