# Supplementary material for: Coxiella burnetii inhibits host cell death by ubiquitination‐induced inactivation of an apoptosis nuclease
Source: mLife. 2026 Aug 27:10.1002/mlf2.70093. Online ahead of print. doi: 10.1002/mlf2.70093 (PMC13519585; doi:10.1002/mlf2.70093)
Supplement: Supplementary file 1 [file MLF2-9999-0-s003.docx]

***Coxiella burnetii*** **inhibits host cell death by ubiquitination-induced inactivation of an apoptosis nuclease** 

Yong Zhang^a^*, Jun Jiao^b^, Shuxin Liu^a^, Jingya Yuan^a^, Siyao Liu^a^, Tao-tao Chen^c^, Songying Ouyang^c^, Jiaqi Fu^a^* and Zhao-Qing Luo^a^*

^a^Department of Respiratory Medicine, Center of Infectious Diseases and Pathogen Biology, Key Laboratory of Organ Regeneration and Transplantation of The Ministry of Education, State Key Laboratory for Diagnosis and Treatment of Severe Zoonotic Infectious Diseases, The First Hospital of Jilin University; Changchun 130021, China 

^b^ State Key Laboratory of Pathogen and Biosecurity, Beijing Institute of Microbiology and Epidemiology, Fengtai, Beijing, China.

^c^ Key Laboratory of Microbial Pathogenesis and Interventions of Fujian Province University, the Key Laboratory of Innate Immune Biology of Fujian Province, Biomedical Research Center of South China, Key Laboratory of OptoElectronic Science and Technology for Medicine of the Ministry of Education, College of Life Sciences, Fujian Normal University, Fuzhou, 350117, China.

*Correspondence: Yong Zhang (zhangybh@jlu.edu.cn); Jiaqi Fu (fujiaqi@jlu.edu.cn); Zhao-Qing Luo (luoz@jlu.edu.cn)

**Supplementary information**

Supplementary Figures 1 to 9 

Supplementary Tables 1 to 2 

**Fig. S1 Identification of E3 ubiquitin ligases from** ***C. burnetii*** **by biochemical reactions using combinations of E2 enzymes.** 

**A.** Each reaction contained 10-μg E1，2-μg E2, 5-μg Biotin-Ub and 500-μl lysates *of* broth-grown *C. burnetii* (2.7 mg/mL). E2 enzymes were used as the follows: A. Ube2A, Ube2B, Ube2C, Ube2D1, Ube2D2;  B. Ube2D3, Ube2D4, Ube2E1, Ube2E2, Ube2E3; C. Ube2F, Ube2G1, Ube2G2, Ube2H, Ube2I; D. Ube2J1, Ube2K, Ube2L3, Ube2L6, Ube2M; E. Ube2N, Ube2Q2, Ube2R1, Ube2R2, Ube2S; F. Ube2T, Ube2V2, Ube2W, Ube2Z, Ube2J2; G. No E1 or E2. The reactions were allowed to proceed for 2 h at 37^o^C before adding 20-μl streptavidin beads. After 3x washes, 4% of the enriched proteins and 5% of reactions were analyzed by detecting biotinylated proteins with streptavidin. The remaining enriched proteins were used to identify potentially ubiquitinated proteins by mass spectrometric analysis. 

**B.** Reversal of ubiquitination catalyzed by CubL1 by the deubiquitinase SdeA_1-200_. Samples of ubiquitination reactions with GST-CubL1 were incubated with SdeA and ubiquitinated CubL1 was probed by immunoblotting with antibodies specific for GST or Ubiquitin. Note that SdeA removed ubiquitination signals when detected by antibodies against either GST or ubiquitin. 

**C.** Reversal of CubL1-mediated ubiquitination by host cell lysates.

Samples of ubiquitination reactions with GST-CubL1 were incubated with cell lysates and ubiquitinated CubL1 was probed by immunoblotting with antibodies specific for GST. Noting that cell lysates removed ubiquitination signals on CubL1.

**Fig. S2 The ligase activity of CubL1 does not need a cysteine residue**

Recombinant GST-CubL1 or its cysteine mutants were used for standard ubiquitination reactions. Ligase activity was assessed by molecular weight shift after immunoblotting with GST-specific antibodies. 

**Fig. S3 Self-ubiquitination is not important for the E3 ligase activity of CubL1**

Reactions containing recombinant GST-CubL1 purified from *E. coli* and other components for ubiquitination were allowed to proceed for 2 h and the products were detected by Coomassie brilliant blue staining after SDS-PAGE. The protein band representing self-modified CubL1 was excised and analyzed to determine the site of ubiquitination by mass spectrometric analysis (A). Two tryptic peptides harboring the Lys-ϵ-Gly-Gly (diGLY) remnant were detected, which revealed that K446 and K467 were ubiquitinated (B). Modification of eIF3g by the CubL1_K446R/K467R_ mutant defective in self-ubiquitination. Ubiquitination reactions containing the indicated components were allowed to proceed for 2 h and self-ubiquitination (upper panel) and ubiquitnation of eIF3g (lower panel) was detected by immunblotting. Note that CubL1_K446R/K467R_ can no longer undergo self-ubiquitination but it retained the ability to ubiquitinate eIF3g (8th lane).

**Fig. S4 Direct interaction of CubL1 with ubiquitin and UbcH5C.** 

Full-length CubL1 or CubL1_3R_ was immobilized on a CM5 chip, followed by the injection of various concentrations of ubiquitin or UbcH5C. The response unit (RU) value and affinities of CubL1 or CubL1_3R_ interactions with Ubiquitin **(A-B)** and UbcH5C **(C)** were determined by a Biacore X100.

**Fig. S5 CubL1 does not detectably impact protein synthesis in mammalian cells** 

5x10^4^ Cells transfected to express GFP, GFP-SidI or GFP-CubL1 for 16 h, cells were fed with L-homopropargylglycine (HPG) for 45 min, proteins incorporated with HPG was labeled with azide-containing Alexa Fluor, all provided by the Click-IT kit. Cell images were acquired using an Olympus IX-83 microscope (left). Note that no new protein synthesis was detected in samples transfected to express GFP-SidI. The expression of the testing proteins was detected by immunoblotting (right).

**Fig. S6 Schematic of Ub-eIF3g and Ub-eIF3g-N purification.** 

To produce ubiquitinated GST-eIF3g, ubiquitination assays were performed in a reaction buffer. Ubiquitinated GST-eIF3g protein was purified by affinity chromatography using Ni^2+^-NTA beads, followed by a glutathione-Sepharose column. Ubiquitinated GST-eIF3g-N protein was isolated via glutathione-Sepharose column purification after caspase-3 cleavage of ubiquitinated GST-eIF3g. Only the key relevant proteins were shown. 

**Fig. S7 Ubiquitin docks into the nuclease active pocket of eIF3g.**

The three-dimensional structural model the complex formed by ubiquitin and CubL1 was produced by molecular modeling. 

**Fig. S8 Eukaryotic deubiquitinase (DUBs) cannot remove ubiquitin from Ub-eIF3g produced by CubL1.**

Samples of ubiquitination reactions with eIF3g were incubated with cell lysates, purified deubiquitinase USP46 or USP5. Ubiquitinated eIF3g was probed by immunoblotting with antibodies specific for Flag. Note that treatment with cell lysates, USP46 or USP5 did not reduce ubiquitination signals associated with Ub-eIF3g. 

**Fig. S9 Inhibition of host cell death by CubL1 extends to late phase of infection**

THP-1 cells infected with the indicated bacterial strains for the indicated time frames were fixed, samples stained with Annexin V-FITC were analyzed by flow cytometry. Quantitative results were also shown in bar graphs. 

**Table S1.** **Substrates of the Dot/Icm transporter**

| **Protein** | **Peptides** | **PSMs** |
| --- | --- | --- |
| UPF0422 protein CBU_0937 OS=Coxiella burnetii (strain RSA 493 / Nine Mile phase I) OX=227377 GN=CBU_0937 PE=3 SV=1 | 20 | 211 |
| Uncharacterized protein OS=Coxiella burnetii (strain RSA 493 / Nine Mile phase I) OX=227377 GN=CBU_0021 PE=4 SV=1 | 36 | 209 |
| Uncharacterized protein OS=Coxiella burnetii (strain RSA 493 / Nine Mile phase I) OX=227377 GN=CBU_2013 PE=4 SV=1 | 18 | 114 |
| Uncharacterized protein OS=Coxiella burnetii (strain RSA 493 / Nine Mile phase I) OX=227377 GN=CBU_1665 PE=4 SV=1 | 3 | 57 |
| Uncharacterized protein CBU_0041 OS=Coxiella burnetii (strain RSA 493 / Nine Mile phase I) OX=227377 GN=CBU_0041 PE=4 SV=1 | 3 | 43 |
| Uncharacterized protein OS=Coxiella burnetii (strain RSA 493 / Nine Mile phase I) OX=227377 GN=CBU_1752 PE=4 SV=1 | 15 | 41 |
| Hypothetical membrane spanning protein OS=Coxiella burnetii (strain RSA 493 / Nine Mile phase I) OX=227377 GN=CBU_1863 PE=4 SV=1 | 12 | 40 |
| Uncharacterized protein OS=Coxiella burnetii (strain RSA 493 / Nine Mile phase I) OX=227377 GN=CBU_1686 PE=4 SV=1 | 10 | 35 |
| 17 kDa common-antigen OS=Coxiella burnetii (strain RSA 493 / Nine Mile phase I) OX=227377 GN=CBU_1425 PE=4 SV=1 | 5 | 27 |
| Uncharacterized protein OS=Coxiella burnetii (strain RSA 493 / Nine Mile phase I) OX=227377 GN=CBUA0013 PE=4 SV=1 | 4 | 19 |
| Uncharacterized protein OS=Coxiella burnetii (strain RSA 493 / Nine Mile phase I) OX=227377 GN=CBU_1751 PE=4 SV=1 | 6 | 18 |
| Uncharacterized protein CBU_1754 OS=Coxiella burnetii (strain RSA 493 / Nine Mile phase I) OX=227377 GN=CBU_1754 PE=1 SV=1 | 3 | 17 |
| Uncharacterized protein OS=Coxiella burnetii (strain RSA 493 / Nine Mile phase I) OX=227377 GN=CBU_1409 PE=4 SV=1 | 2 | 17 |
| Putative ankyrin repeat protein CBU_0781 OS=Coxiella burnetii (strain RSA 493 / Nine Mile phase I) OX=227377 GN=CBU_0781 PE=4 SV=1 | 1 | 15 |
| Fic family protein OS=Coxiella burnetii (strain RSA 493 / Nine Mile phase I) OX=227377 GN=CBU_2078 PE=4 SV=1 | 4 | 15 |
| Tetratricopeptide repeat family protein OS=Coxiella burnetii (strain RSA 493 / Nine Mile phase I) OX=227377 GN=CBU_1457 PE=4 SV=1 | 5 | 13 |
| Uncharacterized protein OS=Coxiella burnetii (strain RSA 493 / Nine Mile phase I) OX=227377 GN=CBUA0023 PE=4 SV=1 | 4 | 12 |
| Probable transcriptional regulatory protein CBU_1566 OS=Coxiella burnetii (strain RSA 493 / Nine Mile phase I) OX=227377 GN=CBU_1566 PE=1 SV=1 | 5 | 11 |
| Hypothetical cytosolic protein OS=Coxiella burnetii (strain RSA 493 / Nine Mile phase I) OX=227377 GN=CBU_1569 PE=4 SV=1 | 5 | 10 |
| Uncharacterized protein OS=Coxiella burnetii (strain RSA 493 / Nine Mile phase I) OX=227377 GN=CBU_2007 PE=4 SV=1 | 5 | 9 |
| Uncharacterized protein OS=Coxiella burnetii (strain RSA 493 / Nine Mile phase I) OX=227377 GN=CBU_1685 PE=4 SV=1 | 3 | 7 |
| Uncharacterized protein OS=Coxiella burnetii (strain RSA 493 / Nine Mile phase I) OX=227377 GN=CBU_1794 PE=4 SV=2 | 6 | 7 |
| Uncharacterized protein OS=Coxiella burnetii (strain RSA 493 / Nine Mile phase I) OX=227377 GN=CBU_1543 PE=4 SV=1 | 2 | 5 |
| Hypothetical cytosolic protein OS=Coxiella burnetii (strain RSA 493 / Nine Mile phase I) OX=227377 GN=CBU_1387 PE=4 SV=1 | 2 | 5 |
| Hypothetical membrane associated protein OS=Coxiella burnetii (strain RSA 493 / Nine Mile phase I) OX=227377 GN=CBU_0534 PE=4 SV=1 | 2 | 5 |
| Hypothetical membrane spanning protein OS=Coxiella burnetii (strain RSA 493 / Nine Mile phase I) OX=227377 GN=CBU_1576 PE=4 SV=1 | 2 | 4 |
| Uncharacterized protein OS=Coxiella burnetii (strain RSA 493 / Nine Mile phase I) OX=227377 GN=CBU_0665 PE=4 SV=1 | 3 | 4 |
| Uncharacterized protein OS=Coxiella burnetii (strain RSA 493 / Nine Mile phase I) OX=227377 GN=CBU_0295 PE=4 SV=1 | 3 | 4 |
| Uncharacterized protein OS=Coxiella burnetii (strain RSA 493 / Nine Mile phase I) OX=227377 GN=CBU_1724 PE=4 SV=1 | 4 | 4 |
| Uncharacterized protein OS=Coxiella burnetii (strain RSA 493 / Nine Mile phase I) OX=227377 GN=CBU_1493 PE=4 SV=2 | 4 | 4 |
| Ankyrin repeat protein OS=Coxiella burnetii (strain RSA 493 / Nine Mile phase I) OX=227377 GN=CBU_0072 PE=4 SV=1 | 2 | 4 |
| Hypothetical membrane spanning protein OS=Coxiella burnetii (strain RSA 493 / Nine Mile phase I) OX=227377 GN=CBU_0077 PE=4 SV=1 | 3 | 3 |
| GatB/YqeY domain protein OS=Coxiella burnetii (strain RSA 493 / Nine Mile phase I) OX=227377 GN=CBU_1594 PE=4 SV=1 | 3 | 3 |
| Ankyrin repeat protein OS=Coxiella burnetii (strain RSA 493 / Nine Mile phase I) OX=227377 GN=CBU_0447 PE=4 SV=1 | 2 | 3 |
| Hypothetical cytosolic protein OS=Coxiella burnetii (strain RSA 493 / Nine Mile phase I) OX=227377 GN=CBU_1676 PE=4 SV=1 | 3 | 3 |
| Uncharacterized protein OS=Coxiella burnetii (strain RSA 493 / Nine Mile phase I) OX=227377 GN=CBUA0020 PE=4 SV=1 | 2 | 3 |
| Hypothetical membrane spanning protein OS=Coxiella burnetii (strain RSA 493 / Nine Mile phase I) OX=227377 GN=CBU_0410 PE=4 SV=1 | 3 | 3 |
| Uncharacterized protein OS=Coxiella burnetii (strain RSA 493 / Nine Mile phase I) OX=227377 GN=CBU_0794 PE=4 SV=1 | 3 | 3 |
| Uncharacterized protein CBU_1818 OS=Coxiella burnetii (strain RSA 493 / Nine Mile phase I) OX=227377 GN=CBU_1818 PE=4 SV=1 | 1 | 2 |
| Uncharacterized protein OS=Coxiella burnetii (strain RSA 493 / Nine Mile phase I) OX=227377 GN=CBU_1790 PE=4 SV=1 | 1 | 2 |
| Uncharacterized protein OS=Coxiella burnetii (strain RSA 493 / Nine Mile phase I) OX=227377 GN=CBU_1639 PE=4 SV=1 | 1 | 2 |
| Uncharacterized protein OS=Coxiella burnetii (strain RSA 493 / Nine Mile phase I) OX=227377 GN=CBU_0425 PE=4 SV=1 | 1 | 2 |
| Hypothetical membrane spanning protein OS=Coxiella burnetii (strain RSA 493 / Nine Mile phase I) OX=227377 GN=CBU_1556 PE=4 SV=1 | 2 | 2 |
| Uncharacterized protein OS=Coxiella burnetii (strain RSA 493 / Nine Mile phase I) OX=227377 GN=CBU_0626 PE=4 SV=1 | 2 | 2 |
| Uncharacterized protein OS=Coxiella burnetii (strain RSA 493 / Nine Mile phase I) OX=227377 GN=CBU_0388 PE=4 SV=1 | 2 | 2 |
| Uncharacterized protein OS=Coxiella burnetii (strain RSA 493 / Nine Mile phase I) OX=227377 GN=CBU_1063 PE=4 SV=1 | 2 | 2 |
| Hypothetical cytosolic protein OS=Coxiella burnetii (strain RSA 493 / Nine Mile phase I) OX=227377 GN=CBU_1198 PE=4 SV=1 | 2 | 2 |
| Ankyrin repeat protein OS=Coxiella burnetii (strain RSA 493 / Nine Mile phase I) OX=227377 GN=CBU_1213 PE=4 SV=1 | 2 | 2 |
| Hypothetical membrane spanning protein OS=Coxiella burnetii (strain RSA 493 / Nine Mile phase I) OX=227377 GN=CBU_1217 PE=4 SV=1 | 2 | 2 |
| Uncharacterized protein CBU_1819 OS=Coxiella burnetii (strain RSA 493 / Nine Mile phase I) OX=227377 GN=CBU_1819 PE=4 SV=1 | 2 | 2 |
| Gluconolactonase OS=Coxiella burnetii (strain RSA 493 / Nine Mile phase I) OX=227377 GN=CBU_1789 PE=4 SV=1 | 1 | 1 |
| Hypothetical membrane spanning protein OS=Coxiella burnetii (strain RSA 493 / Nine Mile phase I) OX=227377 GN=CBU_0635 PE=4 SV=1 | 1 | 1 |
| Uncharacterized protein OS=Coxiella burnetii (strain RSA 493 / Nine Mile phase I) OX=227377 GN=CBU_1607 PE=4 SV=1 | 1 | 1 |
| Uncharacterized protein CBUA0006 OS=Coxiella burnetii (strain RSA 493 / Nine Mile phase I) OX=227377 GN=CBUA0006 PE=4 SV=1 | 1 | 1 |
| Hypothetical cytosolic protein OS=Coxiella burnetii (strain RSA 493 / Nine Mile phase I) OX=227377 GN=CBU_0881 PE=4 SV=1 | 1 | 1 |
| Serine/threonine protein kinase domain protein OS=Coxiella burnetii (strain RSA 493 / Nine Mile phase I) OX=227377 GN=CBU_0175 PE=4 SV=1 | 1 | 1 |
| Hypothetical cytosolic protein OS=Coxiella burnetii (strain RSA 493 / Nine Mile phase I) OX=227377 GN=CBU_1314 PE=4 SV=2 | 1 | 1 |
| Uncharacterized protein OS=Coxiella burnetii (strain RSA 493 / Nine Mile phase I) OX=227377 GN=CBU_1178 PE=4 SV=1 | 1 | 1 |
| Hypothetical cytosolic protein OS=Coxiella burnetii (strain RSA 493 / Nine Mile phase I) OX=227377 GN=CBU_2016 PE=4 SV=2 | 1 | 1 |

**Table S2. Bacterial Strains, plasmid and primers used in this study** 

**Bacterial Strains used in this study**

| **Designation** | **SOURCE** | **IDENTIFIER** |
| --- | --- | --- |
| C. burnetii Nine Mile phase II (RSA493) |  | N/A |
| *icmD::Tn* | This paper | N/A |
| *cubL1::Tn* | This paper | N/A |
| *cubL1::Tn*(pCubL1) | This paper | N/A |
| *cubL1::Tn*(pCubL1_3R_) | This paper | N/A |

**Plasmids used in this study** 

| **name** | **source** |
| --- | --- |
| pJB-Kan-P1169-3xFLAG | (Beare et al, 2012) |
| pJB-Flag-TEM1-EliC1 | This paper |
| pEGFPC1-EliC1 | This paper |
| pCMV4xFlag | (Li and Qiu et al, 2020) |
| pET28a | Invitrogen |
| pET28a-EliC1 | This paper |
| pGEX6p-1-EliC1 | This paper |
| pGEX6p-1-EliC1_C22A_ | This paper |
| pGEX6p-1-EliC1_C69A_ | This paper |
| pGEX6p-1-EliC1_C220A_ | This paper |
| pGEX6p-1-EliC1_C232A_ | This paper |
| pGEX6p-1-EliC1_C250A_ | This paper |
| pGEX6p-1-EliC1_C314A_ | This paper |
| pGEX6p-1-EliC1_C372A_ | This paper |
| pGEX6p-1-EliC1_E164R_ | This paper |
| pGEX6p-1-EliC1_E166R_ | This paper |
| pGEX6p-1-EliC1_E164R/E166R_ | This paper |
| pGEX6p-1-EliC1_Q196R_ | This paper |
| pGEX6p-1-EliC1_E228R_ | This paper |
| pGEX6p-1-EliC1_Q225R/E228R_ | This paper |
| pGEX6p-1-EliC1_Q196R/Q225R/E228R_ | This paper |
| pET28a-Uniquitin | This paper |
| pET28a-Uniquitin(K6A) | This paper |
| pET28a-Uniquitin(K11A) | This paper |
| pET28a-Uniquitin(K27A) | This paper |
| pET28a-Uniquitin(K29A) | This paper |
| pET28a-Uniquitin(K33A) | This paper |
| pET28a-Uniquitin(K48A) | This paper |
| pET28a-Uniquitin(K63A) | This paper |
| pET28a-Uniquitin(KO) | This paper |
| pET28a-Uniquitin(G75A,G76A) | This paper |
| pET28a-UbcH5c | This paper |
| pET28a-UbcH7 | This paper |
| pET28a-E1 | This paper |
| pET28a-eIF3g | This paper |
| pET28a-eIF3g(K78R) | This paper |
| pET28a-eIF3g1-230 | This paper |
| pET28a-eIF3g1-230 (K78R) | This paper |
| pCMV | Invitrogen |
| pJB-Flag-TEM1-EliC1 | This paper |
| pCMV-Flag-EliC1 | This paper |
| pCMV-Flag-eIF3g | This paper |
| pCMV-Flag-eIF3g(K78R) | This paper |
| pCMV-Flag-eIF3g1-230 | This paper |
| pCMV-Flag-eIF3g1-230 (K78R) | This paper |
| pJB-Flag-EliC1 | This paper |
| pCMV-Flag-Cbu1576 | This paper |
| pCMV-Flag-Cbu1794 | This paper |
| pCMV-Flag-Cbu1724 | This paper |
| pCMV-Flag-Cbu1556 | This paper |
| pCMV-Flag-Cbu1569 | This paper |
| pQE30-SdeA_(1-200)_ | This paper |

**Reference:**

[1] Beare PA. Genetic manipulation of Coxiella burnetii. Advances in experimental medicine and biology 2012;984:249-71.

[2] Li G, Liu H, Luo ZQ, Qiu J. Modulation of phagosome phosphoinositide dynamics by a Legionella phosphoinositide 3-kinase. EMBO reports 2021;22:e51163.

**Primers used in this study**

| Primer name | Sequence (Restriction enzyme sites are underlined) | Note |
| --- | --- | --- |
| EliC1-F  WT | CGCGGATCCTTGAGGTACAAGCGACACATG | EliC1 5’ BamH I |
| EliC1-R  WT | CTAGTCGACTTAAAAAGTAAAGGATTGTTTAGAG | EliC1 3’ Sal I |
| EliC1-F | CGCGGATCCATGAGATATAAGAGGCACATGGA | EliC1 5’ BamH I |
| EliC1-R | CTAGTCGACGTGGTGGTGGTGGTGGTGAAAGGTAAAGCTCTGCTTGC | EliC1 3’ Sal I |
| EliC1_C22A_-F | CTCACGACTTTTGCGAAAGGCCTCAATAGCGAAGTCAATG | EliC1 mutant C22A |
| EliC1_C22A_-R | CATTGACTTCGCTATTGAGGCCTTTCGCAAAAGTCGTGAG |  |
| EliC1_C69A_-F | GGTAATGTAGTTGAAGTAATTCACAGCGTCTTCATGCGGTTTATCATTGAAA | EliC1 mutant C69A |
| EliC1_C69A_-R | TTTCAATGATAAACCGCATGAAGACGCTGTGAATTACTTCAACTACATTACC |  |
| EliC1_C220A_-F | GATTTGATACTTATTACCGGCCACGGCGAACAGACGCGC | EliC1 mutant C220A |
| EliC1_C220A_-R | GCGCGTCTGTTCGCCGTGGCCGGTAATAAGTATCAAATC |  |
| EliC1_C232A_-F | GAACAGCACCGCTTCATAAGCGCGCTTGGCTTCTTTGATT | EliC1 mutant C232A |
| EliC1_C232A_-R | AATCAAAGAAGCCAAGCGCGCTTATGAAGCGGTGCTGTTC |  |
| EliC1_C250A_-F | TTCTCATGCTCCAGAAAGGCGGCAAAGCGGTAGATTGC | EliC1 mutant C250A |
| EliC1_C250A_-R | GCAATCTACCGCTTTGCCGCCTTTCTGGAGCATGAGAA |  |
| EliC1_C314A_-F | TTTTCGATCGCCTCAATGGCATATTCGATCGCCTTGCCG | EliC1 mutant C314A |
| EliC1_C314A_-R | CGGCAAGGCGATCGAATATGCCATTGAGGCGATCGAAAA |  |
| EliC1_C372A_-F | GTAGTTTTTCAGGCTGTTGGCGTGCTTGATCGCCAATGGC | EliC1 mutant C372A |
| EliC1_C372A_-R | GCCATTGGCGATCAAGCACGCCAACAGCCTGAAAAACTAC |  |
| EliC1_E164R_-F | TTCTCCAGCTCTTCTTTCCTGAGAGAGTGGTTGGCAGC | EliC1 mutant E164R |
| EliC1_E164R_-R | GCTGCCAACCACTCTCTCAGGAAAGAAGAGCTGGAGAA |  |
| EliC1_E166R_-F | CGTTCTCCAGCTCTCTTTTCTCGAGAGAGTGGTTGGCAG | EliC1 mutant E166R |
| EliC1_E166R_-R | CTGCCAACCACTCTCTCGAGAAAAGAGAGCTGGAGAACG |  |
| EliC1_E164R/E166R_-F | GTCTTAGCGTTCTCCAGCTCTCTTTTCCTGAGAGAGTGGTTGGCAGCCAA | EliC1 mutant E164R/E166R |
| EliC1_E164R/E166R_-R | TTGGCTGCCAACCACTCTCTCAGGAAAAGAGAGCTGGAGAACGCTAAGAC |  |
| EliC1_Q196R_-F | ACAGTGGCGTAAGATTCCGACGTTGATACAGGAAG | EliC1 mutant Q196R |
| EliC1_Q196R_-R | CTTCCTGTATCAACGTCGGAATCTTACGCCACTGT |  |
| EliC1_Q225R_-F | GCTTGGCTTCTTTGATTCTATACTTATTACCGCACACGGCG | EliC1 mutant Q225R |
| EliC1_Q225R_-R | CGCCGTGTGCGGTAATAAGTATAGAATCAAAGAAGCCAAGC |  |
| EliC1_E228R_-F | TAACAGCGCTTGGCTCTTTTGATTTGATACTTATTACCGCACACGG | EliC1 mutant E228R |
| EliC1_E228R_-R | CCGTGTGCGGTAATAAGTATCAAATCAAAAGAGCCAAGCGCTGTTA |  |
| EliC1_Q225R/E228R_-F | TAACAGCGCTTGGCTCTTTTGATTCTATACTTATTACCGCACACGGCGAACAGAC | EliC1 mutant Q225R/E228R |
| EliC1_Q225R/E228R_-R | GTCTGTTCGCCGTGTGCGGTAATAAGTATAGAATCAAAAGAGCCAAGCGCTGTTA |  |
| eIF3g-F | CTGAGATCTATGCCTACTGGAGACTTTGATTC | eIF3g 5’ BglⅡ |
| eIF3g-R | CTGGTCGACTTAGTTGGTGGACGGCTTGGC | eIF3g 3’ Sal I |
| HSP90α-F | CTGGGATCCATGCCTGAGGAAACCCA | HSP90α 5’ BamH I |
| HSP90α-R | CTGGTCGACTTAGTCTACTTCTTCCATGCGT | HSP90α 3’ Sal I |
| MYH9-F | CTGGGATCCATGGCACAGCAAGCTGCC | MYH9 5’ BamH I |
| MYH9-R | CTGGTCGACTTATTCGGCAGGTTTGGCCT | MYH9 3’ Sal I |
| P1169 Promoter-F | CTGCTCGAGATGGCTTCGTTTCGCAGCG | P1169 Promoter 5’ Xho I |
| P1169 Promoter-R | CTGGAGCTCCCTCCTTCATGAAGGATTAA | P1169 Promoter 3’ Sac I |
| TEM1-F | GTCGTCGACCCAGAAACGCTGGTGAAAGT | TEM1 5’ Sal I |
| TEM1-R | CTTGGATCCTTACCAATGCTTAATCAGTGAG | TEM1 3’ BamH I |
| Com1-F | AAAACCTCCGCGTTGTCTTCA | Quantify the amount of C. burnetii genomic |
| Com1-R | GCTAATGATACTTTGGCAGCGTATTG |  |
| Com1-probe | AGAACTGCCCATTTTTGGCGGCCA | 5`6-FAM，3`BHQ1 |
| CBU1794-F | CTGGGATCCATGGAGGCTGATATCATG | CBU1794 5’ BamH I |
| CBU1794-R | CTGGTCGACCTATCGATGACTCGTTAA | CBU1794 3’ Sal I |
| CBU1576-F | CTGGGATCCATGCGTCCAGAACATAAA | CBU1576 5’ BamH I |
| CBU1576-R | CTGCTCGAGCTAATGTTTCATCGAATA | CBU1576 3’ Xho I |
| CBU1724-F | CTGGGATCCATGACTCGGCAGATCAAT | CBU1724 5’ BamH I |
| CBU1724-R | CTGGTCGACCTATTTCAGGCCGGGGGG | CBU1724 3’ Sal I |
| CBU1556-F | CTGGGATCCATGCCTTCTGATAGAAAC | CBU1556 5’ BamH I |
| CBU1556-R | CTGGTCGACCTAAACGGGAGCATAATT | CBU1556 3’ Sal I |
| CBU1569-F | CTGGGATCCATGCCTATTACCAGCTTA | CBU1569 5’ BamH I |
| CBU1569-R | CTGGTCGACTTAATTGTTATTTCGAGG | CBU1569 3’ Sal I |
